# Investigating Stable Low-Energy Gallium Oxide (Ga_2_O_3_) Polytypes: Insights into Electronic and Optical Properties from First Principles

**DOI:** 10.1021/acsomega.3c10192

**Published:** 2024-03-26

**Authors:** Arthi Devamanoharan, Vishnukanthan Venkatachalapathy, Vasu Veerapandy, Ponniah Vajeeston

**Affiliations:** † School of Physics, Madurai Kamaraj University, Madurai 625021, India; ‡ Department of Physics/Centre for Materials Science and Nanotechnology, University of Oslo, P.O. Box 1048 Blindern, Oslo NO-0316, Norway; § Department of Materials Science, National Research Nuclear University “MEPhI”, 31 Kashirskoe Sh., Moscow 115409, Russian Federation; ∥ Center for Materials Science and Nanotechnology, University of Oslo, Oslo 0371, Norway

## Abstract

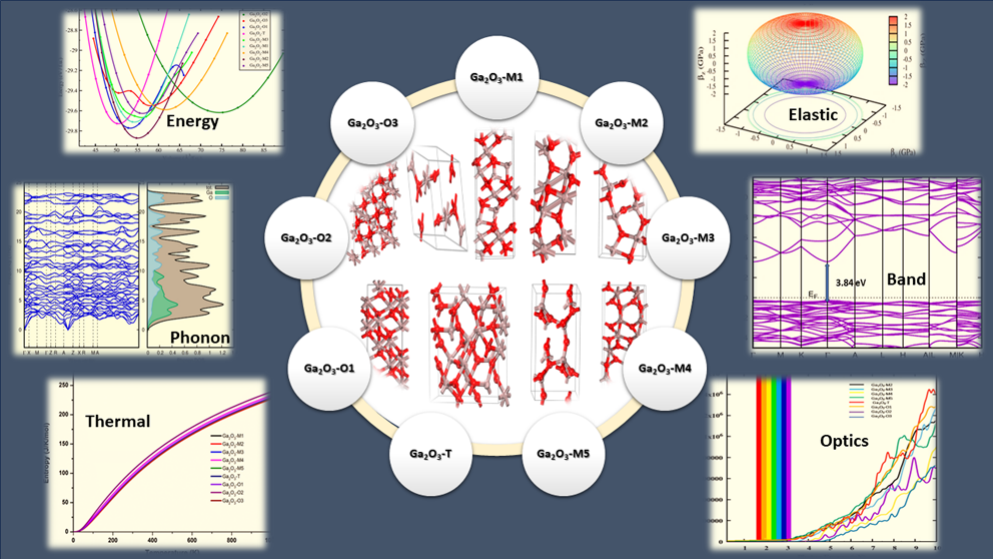

This study provides a comprehensive analysis of the electronic and optical properties of low-energy gallium oxide (Ga_2_O_3_) polytypes not considered earlier. Among these polytypes, the monoclinic structure (β-Ga_2_O_3_) holds significant relevance for both research and practical applications due to its superior stability under typical conditions. The primary aim of this research is to identify new and stable Ga_2_O_3_ polytypes that may exist under zero-temperature and zero-pressure conditions. To achieve this objective, we employ the VASP code to investigate electrical and optical properties, as well as stability assessments. Additionally, we examine phonon and thermal properties, including heat capacity, for all polytypes. This study also encompasses the computation of full elastic tensors and elastic moduli for all polytypes at 0 K, with Poisson’s and Pugh’s ratios confirming their ductile nature. Furthermore, we present the first ever report on the Raman- and infrared (IR)-active modes of these stable Ga_2_O_3_ polytypes. Our findings reveal that these mechanically and dynamically stable Ga_2_O_3_ polytypes exhibit semiconductive properties, as evidenced by electronic band structure investigations. This research offers valuable insights into the optical characteristics of Ga_2_O_3_ polytypes with potential applications spanning various fields.

## 1 Introduction

Gallium oxide (Ga_2_O_3_) holds the distinction of possessing the widest band gap among transparent semiconducting oxides. This material boasts remarkable translucency, extending into the UV–C spectral range.^1^ Its band gap measures approximately 4.9 eV, and it displays polymorphism. Depending on the preparation conditions, Ga_2_O_3_ can take on five distinct crystalline structures: monoclinic (β), trigonal (corundum) (α), spinel-type (γ), cubic (δ), and hexagonal (ε) forms.^2−4^ Of these, the β polytype has gained the most attention due to its superior thermal and chemical stability in typical situations.^5^ Ga_2_O_3_ shows promise in a variety of fields, including solar-blind UV detectors, flat-panel displays, high-temperature stable gas sensors, passivation coating, and antireflection coating on GaAs, and is appropriate for use in optoelectronic devices as it demonstrates transparent conducting qualities.^6−9^ The monoclinic polytype remains stable under normal pressure and temperature conditions but can transform into other polytypes at higher pressures or temperatures.^10^ It undergoes a β-Ga_2_O_3_ to α-Ga_2_O_3_ transition at 4.4 GPa and 1000 °C.^11^ According to He et al., the α-Ga_2_O_3_ to β-Ga_2_O_3_ transformation takes place at 573 K under wet conditions, while the ε-Ga_2_O_3_ to β-Ga_2_O_3_ transformation occurs at 1143 K under dry atmosphere conditions.^12^

Ga_2_O_3_ has also emerged as a promising contender in solar-blind photodetectors due to its wide band gap. Ga_2_O_3_-based photodetectors have a cutoff wavelength of 256–280 nm, which efficiently satisfies the criteria for deep ultraviolet detection and power electronics.^13,14^ Additionally, the distinctive conduction electron spin magnetism of Ga_2_O_3_ exhibits an unusual memory effect over a wide temperature range, from 4 K to ambient temperature.^15^ Ga_2_O_3_ shows great potential in applications such as Schottky barrier diodes and field-effect transistors. Also, it boasts a theoretical breakdown field that can reach up to 8 MV/cm, a characteristic that has gathered attention in the ultrahigh-power device market, better than SiC and GaN-based electronics.^16^ Thin films of polycrystalline β-Ga_2_O_3_ containing oxygen vacancies have been recognized as sensors for diverse gases, including H_2_, CH_4_, CO, and O_2_, with changes in electrical conductivity upon adsorption.^17^

The abundance of theoretical and experimental studies primarily concentrate on its monoclinic β-Ga_2_O_3_ phase, with very little attention paid to the other phases. Litimein et al. conducted calculations on the electronic structure and optical properties of β-Ga_2_O_3_ using the full-potential linearized augmented plane wave approach.^18^ Exploring the structural attributes of five Ga_2_O_3_ polymorphs, Yoshioka et al. employed the pseudopotential method in conjunction with the GGA functional.^6^ He et al. investigated the structural, electronic, and optical characteristics of this compound in its monoclinic and hexagonal phases, utilizing the linear combination of the atomic orbital method along with Becke’s three-parameter hybrid exchange functional (B3LYP).^19^

In this paper, our focus is directed toward the investigation of the structural, electronic, and optical properties of stable low-energy Ga_2_O_3_ polytypes under zero-temperature and zero-pressure conditions. As per our present understanding, there is a lack of previous documentation regarding comprehensive full-potential calculations concerning these specific properties across the 9 low-energy polytypes considered.

This study of low-energy Ga_2_O_3_ polytypes is categorized into five major sections: structural properties, phonon properties, thermal properties, mechanical properties, and electrical and optical properties. The structural properties encompass factors like crystal allotropes and formation energy. The phonon properties segment focuses on the dynamical stability, phonon density of states, irreducible representations for Raman and infrared (IR) phonons, as well as theoretical values for phonon mode frequencies. The thermal properties section discusses parameters such as Debye temperature, specific heat, entropy, and internal energy. The electronic properties include energy gap and density of states. The optical properties include dielectric function, refractive index, absorption coefficient, reflectivity, and energy loss function.

## 2 Results and Discussion

### 2.1 Structural Parameters

The process of choosing input structures of Ga_2_O_3_ from the ICSD database for the A_2_X_3_ composition demands considerable effort and involves substantial computational resources, as outlined in our prior study.^20^ Through the utilization of energy–volume curve fitting, the process of identifying polytypes with low energy becomes viable. We selected only the nine Ga_2_O_3_ polytypes with lower energy levels out of the available options. These chosen configurations exhibit energy deviations of merely 400 meV when compared to the lowest-energy structure. Additionally, we excluded polytypes with higher energy levels as well as those with minor differences in *c*/*a* values (<3%). The outcome of this selection reaffirms the predominance of the monoclinic system as the most stable, while the energetically closest polytypes belong to the orthorhombic and trigonal systems. Among this set of 9 polytypes, they crystallize into three distinct crystal structures: five exhibit a monoclinic configuration, one trigonal system, and three adopt an orthorhombic arrangement. The Ga_2_O_3_ polytypes, namely, Ga_2_O_3_-M1, -M2, -M3, -M4, and -M5, exhibit distinct monoclinic crystal structures characterized by space groups *C*2/*c, C*2/*m*, *Cm*, *P2*_1_/*c*, and *P2*_1_/*c* respectively. The lattice parameters for all of these polytypes are provided in Table 1, based on their equilibrium state. Åhman et al. have provided a comprehensive analysis of the crystal structure of β-Ga_2_O_3_. While the lattice constants of the monoclinic polytypes do not align precisely with the experimentally confirmed β-Ga_2_O_3_ phase (*a* = 12.21 Å, *b* = 3.04 Å, *c* = 5.80 Å), the *c*/*a* ratios of Ga_2_O_3_-M2 and β-Ga_2_O_3_ are remarkably close (<1%).^21^ The Ga_2_O_3_-T polytype features a rhombohedral lattice structure within the trigonal crystal system, exhibiting similarity to the stable phase of α-Al_2_O_3_, also referred to as corundum. The calculated lattice parameters are *a* = 5.043 Å and *c* = 13.649 Å, which closely correspond to the experimental values of α-Ga_2_O_3_ (*a* = 4.98 Å, *c* = 13.43 Å) reported by Cheng et al.^22^ The work by Yoshioka et al. revealed an orthorhombic structure corresponding to κ-Ga_2_O_3_, with lattice parameters *a* = 5.1 Å, *b* = 8.8 Å, and *c* = 9.4 Å.^6^ Among the orthorhombic polytypes, Ga_2_O_3_-O1, featuring the space group *Pna*2_1_, exhibits lattice parameters (*a* = 5.102 Å, *b* = 8.808 Å, and *c* = 9.399 Å) that closely resemble the previously reported findings. Other low-energy polytypes Ga_2_O_3_-M1, -M3, -M4, -M5, and Ga_2_O_3_-O2, -O3 incorporating new position coordinates have been included in the assessment of stability for the first time. The remaining structural parameters, including Wyckoff positions and their corresponding sites, are detailed in the Supporting Information (Table S1). The optimized structures of the Ga_2_O_3_ polytypes are depicted in Figure 2.

**Table 1:** Optimized Equilibrium Lattice Parameters, Equilibrium Energy, Equilibrium Volume, and Formation Energy of All Ga_2_O_3_ Polytypes with the Existing Experimental Values

polytype name	number of formula units	unit cell constants (Å)	minimum energy (eV/f.u.)	equilibrium volume (Å^3^/f.u.)	formation energy (eV/f.u.)
space group					
Ga_2_O_3_-M1	16	*a* = 7.6140	–29.71	53.79	–11.53
[mp-32570]		*b* = 7.5801			
*C*2/*c* (no.15)		*c* = 11.7175			
Ga_2_O_3_-M2	2	*a* = 9.868; 12.214^21^	–29.87	54.75	–11.69
[mp-34243]		*b* = 2.448; 3.037^21^	–29.85		–11.30^23^
*C*2/*m* (no.12)		*c* = 4.667; 5.798^21^			
Ga_2_O_3_-M3	8	*a* = 6.0435	–29.66	57.04	–11.48
[mp-685036]		*b* = 5.9826			
*Cm* (no. 8)		*c* = 14.6001			
Ga_2_O_3_-M4	4	*a* = 5.6297	–29.59	61.04	–11.41
[mp-754531]		*b* = 8.6198			
*P*2_1_/*c* (no.14)		*c* = 5.7526			
Ga_2_O_3_-M5	4	*a* = 3.3431	–29.62	55.49	–11.45
[mp-755066]		*b* = 12.5608			
*P*2_1_/*c* (no.14)		*c* = 5.4965			
Ga_2_O_3_-T	6	*a* = 5.043; 5.059;^6^ 4.983^22^	–29.73	50.11	–11.55
[mp-22323]					
*R*3̅*c* (no.167)		*c* = 13.649; 13.618;^6^ 13.433^22^			
Ga_2_O_3_-O1	8	*a* = 5.102; 5.120^6^	–29.77	52.79	–11.59
[mp-2254]		*b* = 8.807; 8.790^6^			
*Pna*2_1_ (no.33)		*c* = 9.399; 9.399^6^			
Ga_2_O_3_-O2	4	*a* = 10.252	–29.62	75.25	–11.44
[mp-754401]		*b* = 5.6704			
*Cmc*2_1_ (no.36)		*c* = 5.1781			
Ga_2_O_3_-O3	8	*a* = 4.7424	–29.54	59.38	–11.54
[mp-1194571]		*b* = 5.4683			
*Pbca* (no.61)		*c* = 13.7386			

The estimation of vital equilibrium parameters, such as the equilibrium energy, equilibrium volume, equilibrium bulk modulus, and its derivative, is made easier by fitting the energy–volume curve. The configuration of the energy–volume curve offers valuable insights into the stability variations among distinct crystal structures. Generally, the state characterized by the lowest energy signifies the utmost stability. Across the spectrum of polytypes, their energy minimum falls within the range of −29.9 to −29.5 eV/f.u.

**Figure 1:**
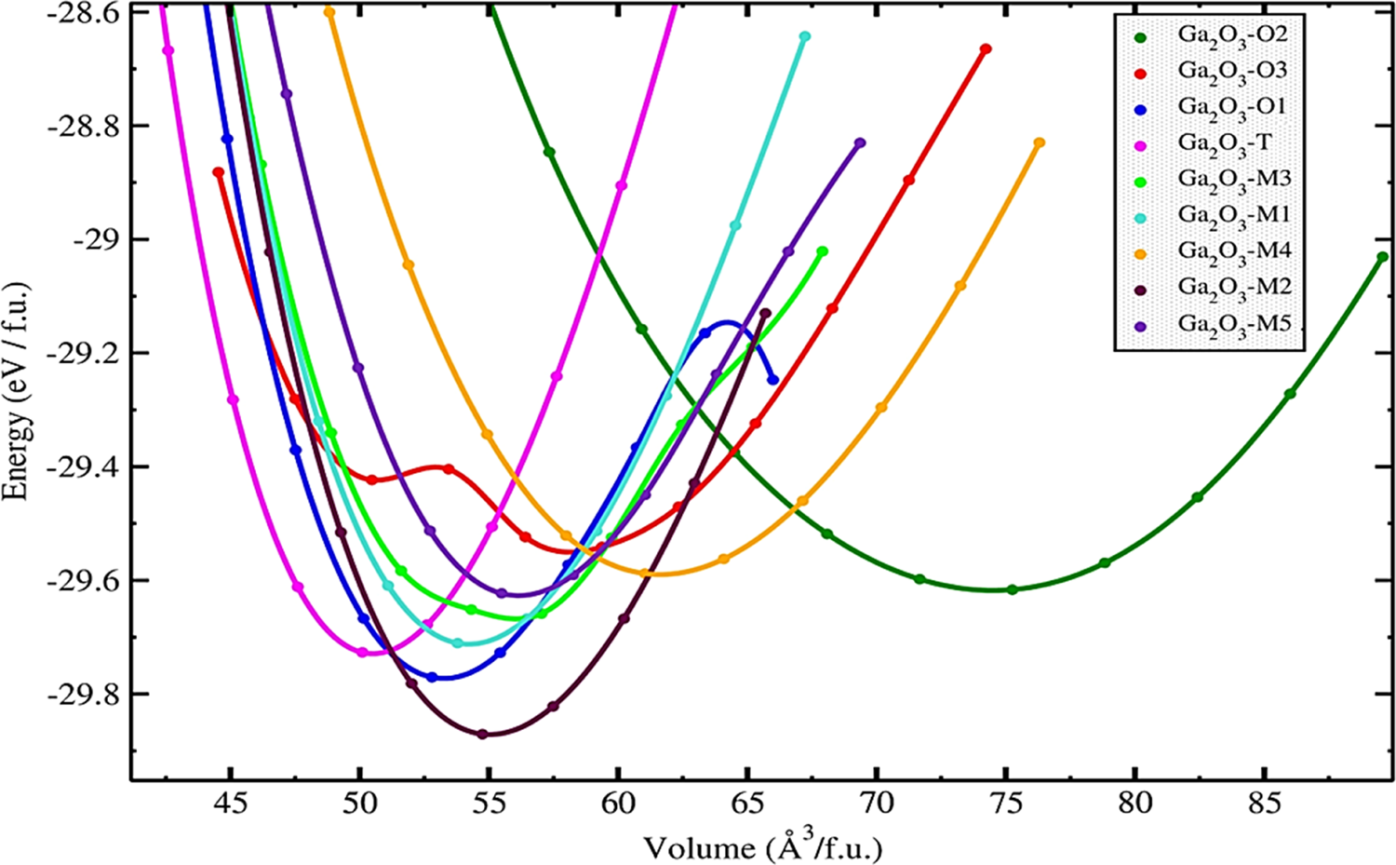
Total energy (*E*) vs volume (*V*) curve for Ga_2_O_3_ polytypes represents all energy–volume values normalized to one formula unit (f.u.).

The polytype Ga_2_O_3_-M2 (−29.87 eV/f.u.) exhibits the highest stability, closely matching the energy level of the experimentally confirmed β-Ga_2_O_3_ phase (−29.86 eV/f.u.).^24^ As depicted in Figure 1, Ga_2_O_3_–O1 (−29.77 eV/f.u.) emerges as energetically stable, positioned closely to the most stable polytype, Ga_2_O_3_-M2. Our study reveals that Ga_2_O_3_-T maintains a stable equilibrium energy of −29.73 eV/f.u. at its equilibrium volume of 50.11 Å^3^, with no signs of potential phase transitions. It is evidenced by the undisturbed nature of its energy–volume fit by that of other polytypes under higher-pressure conditions. The equilibrium volumes of Ga_2_O_3_-M1 and Ga_2_O_3_-M5 polytypes closely approximate that of Ga_2_O_3_-M2. Ga_2_O_3_–O2 exhibits higher total energies exceeding −29.7 eV/f.u. and its equilibrium volume greater than 75 Å^3^/f.u., suggesting comparatively lesser energetic stability within the polytypes considered. The graphical representation in Figure 1 illustrates that, under lower volume and higher-pressure conditions, Ga_2_O_3_-M2 could transform alternative crystal systems characterized by space groups *C*2/*c*, *P*2_1_/*c*, and *Pbca*. Based on the energy–volume curve, we have computed the equilibrium lattice parameters and equilibrium volume for each polytype per formula unit (f.u.), and these findings are summarized in Table 1.

In the context of formation energy (Δ*H*) calculation, gallium is treated as a bulk material [mp-1184502, *C*2/*m*], while the oxygen molecule is positioned at the center of a cubic box with a lattice parameter of 25 Å. The calculated Δ*H* values exhibit negativity across all polytypes, indicating their stability (Table 1). Ga_2_O_3_-M2 exhibits a formation energy of −11.69 eV/f.u. which is 3.45% less compared to the experimental value for β-Ga_2_O_3_ (Δ*H* = −11.30 eV/f.u.) reported by Barin et al.^23^ Moreover, these findings provide further confirmation that Ga_2_O_3_-M2 (−11.69 eV/f.u.) stands as the most stable, while Ga_2_O_3_-M4 (−11.41 eV/f.u.) emerges as the least stable among the polytypes considered. A structure positioned on the convex hull with its formation enthalpy is regarded as thermodynamically stable and experimentally synthesizable. In the case of the Ga_2_O_3_ system, the most stable structure is β-Ga_2_O_3_ and the calculated value of Δ*H* is −2.29 eV/atom (or) – 11.45 eV/f.u. as reported by the study of Banerjee et al.,^25^ whereas Δ*H* values of Ga_2_O_3_ polytypes in our study (−11.69 to −11.41 eV/f.u.) are close to the above-reported value of Δ*H*.

**Figure 2:**
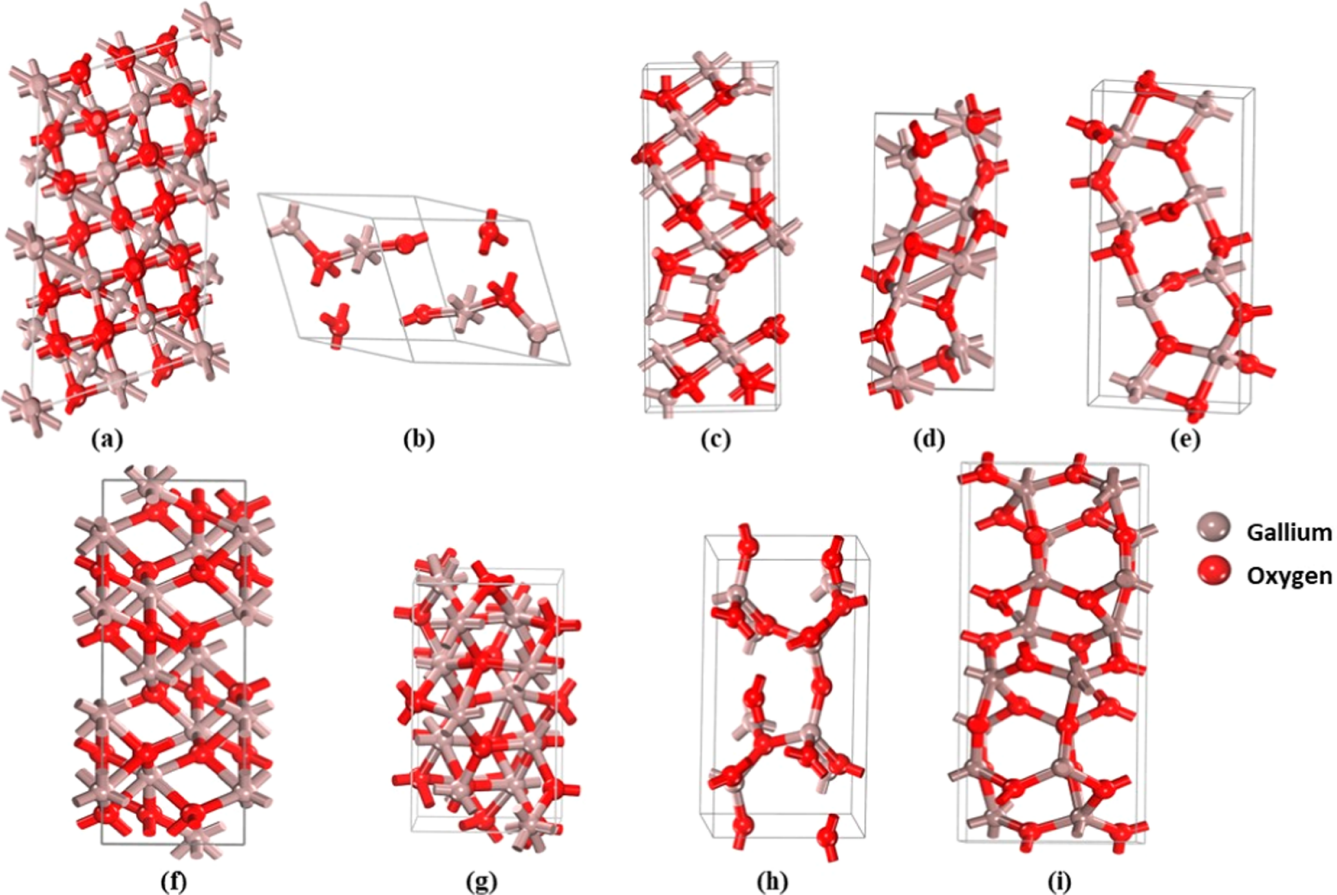
Optimized crystal structure of polytypes: (a) Ga_2_O_3_-M1, (b) Ga_2_O_3_-M2, (c) Ga_2_O_3_-M3, (d) Ga_2_O_3_-M4, (e) Ga_2_O_3_-M5, (f) Ga_2_O_3_-T, (g) Ga_2_O_3_–O1, (h) Ga_2_O_3_–O2, and (i) Ga_2_O_3_–O3.

One of the monoclinic polytypes Ga_2_O_3_-M4 crystallizes within the *P*2_1_/*c* space group (Figure 2). The crystal structure showcases two nonequivalent Ga sites. In the initial Ga site, Ga(1) participates in GaO_5_ trigonal bipyramids through connections with one equivalent of a single O(3), two equivalent O(1), and two equivalent O(2) atoms. In the subsequent Ga site, Ga(2) forms GaO_4_ tetrahedra by bonding with one O(1), one O(2), and two equivalent O(3) atoms. The structure also comprises three distinct O sites. In the first O site, O(1) adopts a distorted trigonal planar configuration, establishing bonds with one Ga(2) and two equivalent Ga(1) atoms. Similarly, the second O site, O(2), displays a distorted T-shaped arrangement, connecting with one Ga(2) atom and two equivalent Ga(1) atoms. Finally, the third O site, O(3), exhibits a distorted trigonal planar geometry, binding with one Ga(1) and two equivalent Ga(2) atoms.

Ga_2_O_3_-T possesses a corundum structure and crystallizes in the trigonal *R*3̅*c* space group (Figure 2). Ga(1) is engaged with six equivalents of O(1) atoms to create a blend of GaO_6_ octahedra, which exhibit distorted corner-, edge-, and face-sharing arrangements. Moreover, O(1) forms bonds with four equivalent Ga(1) atoms, adopting a distorted trigonal pyramidal configuration.

Ga_2_O_3_-O2 adopts an orthorhombic *Cmc*2_1_ space group for its crystal structure. Ga(1) forms bonds with one atom of the O(2) and three equivalent atoms of the O(1) atom, resulting in corner-sharing GaO_4_ tetrahedra. The crystal structure presents two distinct O sites. In the first O site, O(1) establishes connections in a trigonal planar arrangement with three equivalent Ga(1) atoms. In contrast, the second O site, O(2), is involved in a bent geometry at an angle of 150°, bonding to two equivalent Ga(1) atoms. Additional structural details for all of the other polytypes are provided in the Supporting Information (page S1).

### 2.2 Dynamical and Thermal Stability

**Figure 3:**
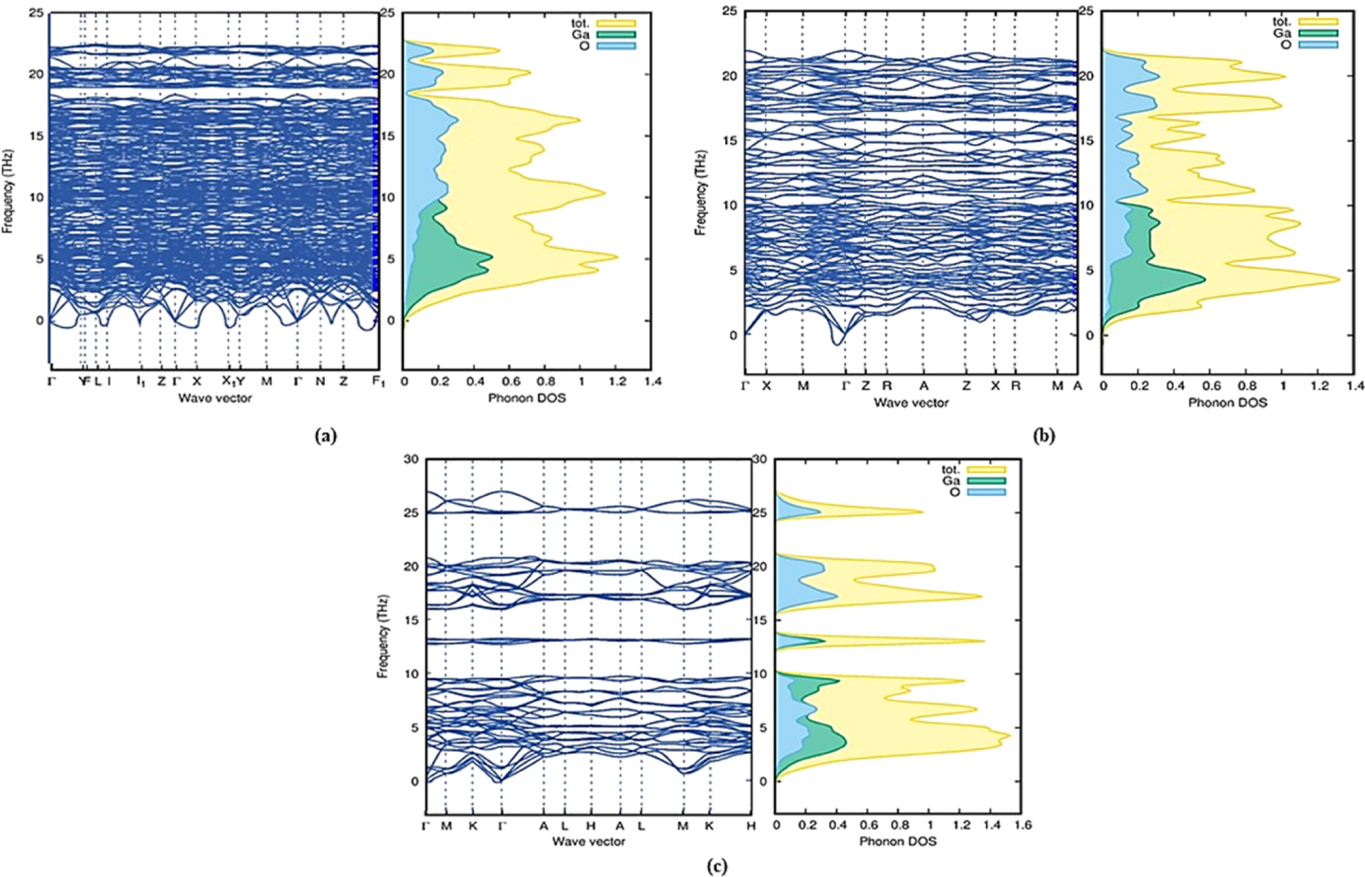
Phonon dispersion curves with PhDOS of Ga_2_O_3_ polytypes: (a) Ga_2_O_3_-M1, (b) Ga_2_O_3_-T, and (c) Ga_2_O_3_–O1.

To provide a comprehensive assessment of dynamical stability, phonon calculations were systematically performed for all polytypes. In the process of investigating the phonon properties, the optimized crystal structure of each polytype served as a starting point. To account for exchange and correlation effects, the generalized gradient approximation as parametrized by (PBE) was applied.^26^ To facilitate phonon calculations, supercell formations were generated using the PHONOPY code.^27^ Subsequently, force sets were generated within this framework. The PHONOPY code was also employed to determine phonon dispersion relations and the phonon density of states. These sequential steps provided a comprehensive understanding of the phonon properties of the investigated polytypes. We further computed phonon dispersion curves along high-symmetry directions for all Ga_2_O_3_ polytypes at their equilibrium volumes in addition to the total phonon density of states. These variations are visually represented in Figures 3 and S1.

All polytypes except Ga_2_O_3_-M1 display positive modes, signifying their dynamical stability. Imaginary frequencies were found at many symmetry points for Ga_2_O_3_-M1, whereas Ga_2_O_3_-M3, Ga_2_O_3_-T, and Ga_2_O_3_-O3 exhibited only a mild imaginary frequency at high-symmetry points Γ. No negative phonon density of states was observed for these polytypes. A review study reported that the presence of these minor imaginary frequencies may be attributed to factors such as numerical calculation issues, inadequately considered parameters when determining force constants or dynamical matrices, or the choice of exchange-correlation parameters.^28^ These issues can be rectified by employing phonon mode-mapping to identify lower-energy structures along imaginary modes or by utilizing an appropriate exchange-correlation functional for DFT calculations.^29,30^ Despite these minor variances, we consider Ga_2_O_3_-M3, -T, and Ga_2_O_3_-O3 as dynamically stable, given the absence of significant negative phonon density of states.

The data from Figures 3 and S1 lead us to the conclusion that, in dynamically stable polytypes, acoustic frequencies are governed by the heavier gallium atoms, while optical frequencies exceeding 10 THz are primarily influenced by the smaller oxygen atoms. However, in all cases, oxygen modes are present in both low- and high-frequency regions. In the polytypes, namely, Ga_2_O_3_-M2, -O2, and Ga_2_O_3_-O3, a distinct phonon band gap exists between the optical and acoustic modes. This separation plays a crucial role in influencing phonon scattering mechanisms and subsequently affects the thermal conductivity of the lattice. Particularly in the case of orthorhombic polytypes Ga_2_O_3_-O2 and Ga_2_O_3_-O3, where a significant energy gap exists between optical and acoustic modes, the ionic bonds binding the atoms exhibit enhanced rigidity. This rigidity contributes to a relatively higher lattice thermal conductivity compared with other polytypes. For Ga_2_O_3_-M2, the presence of a narrower phonon band gap indicates diminished lattice thermal conductivity and an elevated rate of scattering processes. The occurrence of negative (imaginary) phonon frequencies in polytype Ga_2_O_3_-M1 at all high-symmetry points signifies a state of dynamical instability. This instability indicates structural changes throughout the lattice, inducing significant alterations in its configuration.

**Figure 4:**
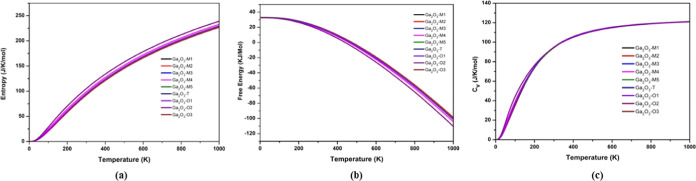
Thermal characteristics: (a) entropy, (b) free energy, and (c) heat capacity as functions of temperature (K) for all polytypes of Ga_2_O_3_.

The thermal characteristics of all Ga_2_O_3_ polytypes, encompassing properties like free energy, entropy, and heat capacity, were investigated. The entire range from 0 to 1000 K was explored to evaluate the thermal attributes across these polytypes. Entropy, which quantifies molecular disorder, undergoes alterations with changing temperature. As the temperature rises, entropy also increases due to a reduction in molecular regularity (Figure 4a). In particular, the entropy of all polytypes exhibited a notable increase, reaching above 200 J/K/mol. As depicted in Figure 4c, all stable polytypes exhibit higher heat capacities (*C*_v_ = 120 J/K/mol), ensuring enhanced stability even at elevated temperatures. Heat capacity reaches the Dulong–Petit limit at higher temperatures, similar to the case of ε-Ga_2_O_3_.^31^

### 2.3 Raman and IR Studies

**Figure 5:**
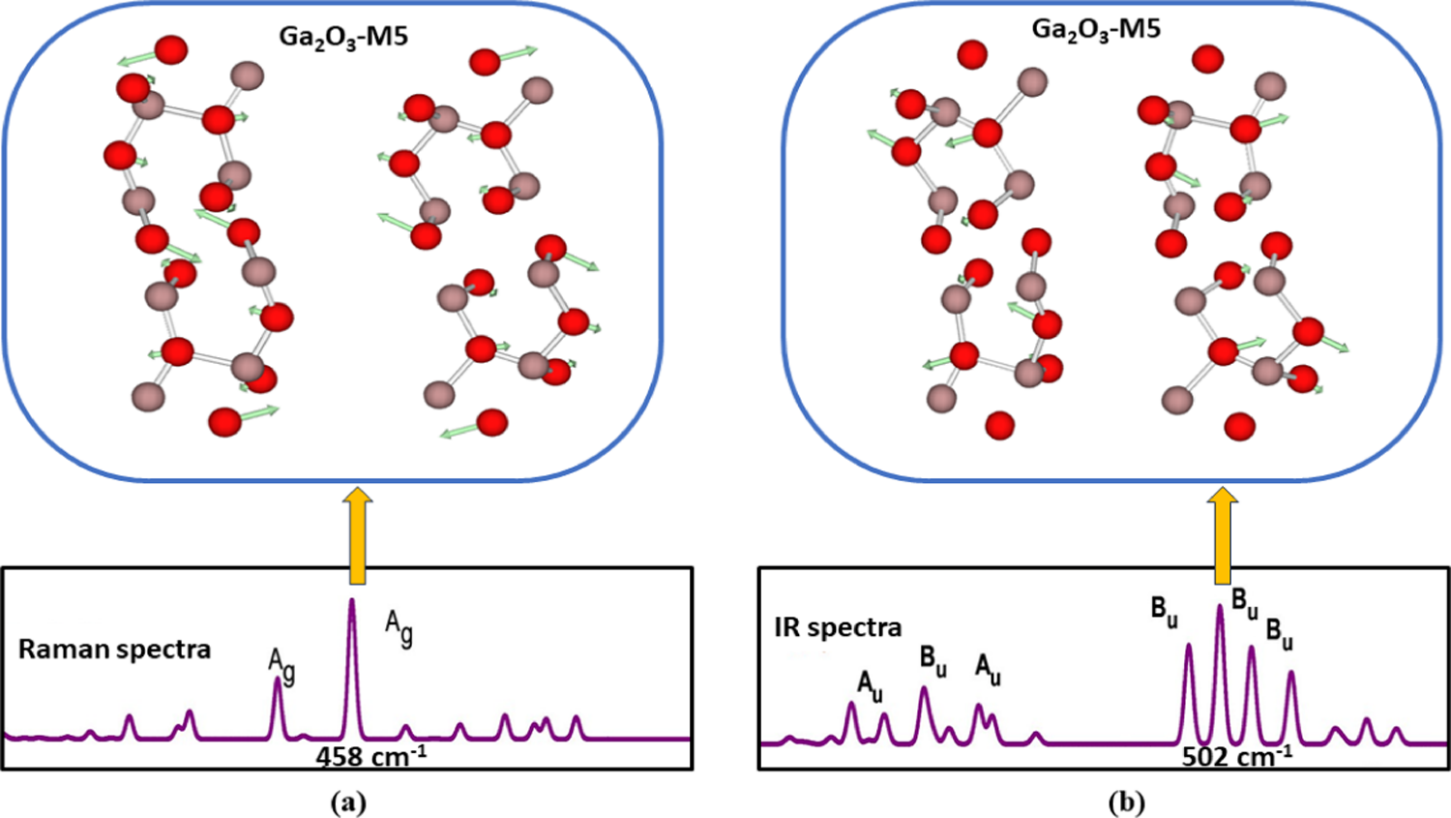
Illustration of the atomic displacements of Ga_2_O_3_-M5 corresponding to (a) the most prominent Raman peak A_g_ (at 458 cm^–1^) and (b) the highest IR peak B_u_ (at 502 cm^–1^) vibration mode.

This research focused on examining the vibrational properties of all of the Ga_2_O_3_ polytypes. Raman spectroscopy serves as a great technique for characterizing the phonon properties at the zone center in bulk crystals.^15^ By utilizing irreducible representations, we can differentiate between the distinct crystalline polytypes of Ga_2_O_3_ based on their Raman and IR activity. The symmetry of all monoclinic Ga_2_O_3_ polytypes aligns with that of the *C*_2*h*_(2/*m)* point group. The irreducible phonon modes at the Brillouin zone center include two Raman-active modes (A_g_, B_g_) and two infrared-active modes (A_u_, B_u_) as depicted in Figure S2.^32^ Raman spectroscopy displays a prominent peak at 418, 584, 370, 410, and 458 cm^–1^ corresponding to the Raman-active A_g_ mode for Ga_2_O_3_-M1, -M2, -M3, -M4, and -M5 polytypes, respectively. The nondegenerate mode A_g_ signifies out-of-plane vibrations of oxygen atoms, which can involve either symmetric stretching or bending relative to the principal axis of symmetry (Figure 5a). A theoretical investigation by Liu et al. finds Raman-active peaks at 104, 317, 360, 474, 600, and 732 cm^–1^ for the β-Ga_2_O_3_ crystal structure.^33^ Interestingly, these peaks closely align with the Raman peaks in this study, listed as 103, 319, 360, 474, 610, and 711 cm^–1^ in Table 2. Turning to the allowed number of IR representations, the Wyckoff positions yield 6, 3, and 6 bands for 4a, 4e, and 8f, respectively. The most prominent band in the spectrum emerges near 314 cm^–1^(A_u_), 526 cm^–1^(B_u_), 330 cm^–1^(A_u_), 608 cm^–1^(B_u_), and 502 cm^–1^(B_u_) for Ga_2_O_3_-M1, -M2, -M3, -M4, and -M5 polytypes, respectively. Numerous faint absorption peaks are also evident, at lower and higher frequencies, as shown in Figure S2 in the Supporting Information.

**Table 2:** Raman- and IR-Active Modes of Ga_2_O_3_ Polytypes

polytype	Raman-active modes (in cm^–1^)		IR-active modes (in cm^–1^)	
Ga_2_O_3_-M1	A_g_	89 128 145 153 159 181 200 226 254 277 293 317 322 339 358 407 418 437 459 479 525 540 547 577 637 652 678 725	A_u_	106 128 141 146 151 179 188 207 220 241 269 314 319 334 340 371 382 392 432 457 471 496 507 539 561 591 626 662 707 736
	B_g_	86 128 130 137 152 172 183 195 208 265 274 292 311 325 342 385 401 416 433 450 460 499 525 538 563 606 638 661 720	B_u_	104 116 132 137 167 172 187 202 216 258 279 309 319 330 344 359 368 377 419 435 443 494 497 516 536 558 581 626 652 713
Ga_2_O_3_-M2	A_g_	103 157 186 296 319 377 436 584 610 711 [104 165 205 317 418 467 600 637 732]^33^	A_u_	145 280 428 627 [146 325 510 668]^33^
	B_g_	107 134 331 449 618 [113 149 360 474 644]^33^	B_u_	194 239 260 331 394 526 643 692 [191 264 283 354 485 625 728 746]^33^
Ga_2_O_3_-M3	A_g_	57 86 98 129 135 156 161 187 219 227 243 263 276 297 320 340 346 370 407 414 436 446 448 455 483 501 517 529 551 591 625 672 689 718	A_u_	27 113 126 138 147 178 248 262 279 298 307 330 343 361 398 443 460 481 535 579 591 623
	B_g_	23 51 71 112 126 139 152 198 249 265 285 304 320 334 346 366 421 448 465 473 502 578 583 620	B_u_	55 104 119 135 142 158 172 196 207 251 260 272 286 287 318 331 361 383 405 411 418 429 445 461 482 498 525 551 590 611 655 673 697
Ga_2_O_3_-M4	A_g_	92 108 133 177 195 257 281 339 369 410 477 519 646 713 731	A_u_	71 110 167 198 247 281 303 337 441 473 559 602 627 758
	B_g_	99 130 163 175 230 254 326 354 363 425 502 556 644 730 773	B_u_	119 140 207 216 238 320 335 469 483 537 608 670 717
Ga_2_O_3_-M5	A_g_	77 108 148 173 196 235 284 296 384 458 512 567 612 640 654	A_u_	68 85 151 188 203 256 296 308 475 533 544 606 627 656
	B_g_	114 130 143 173 217 232 291 330 410 463 541 576 642 652 683	B_u_	135 144 170 216 249 271 345 502 529 563 599 626 652
Ga_2_O_3_-T	A_1g_	201 525 [218 574]^34^	A_2u_	251 503 [271 546]^34^
	^2^E_g_	223 265 309 403 636 [241 286 328 431 687]^34^	^2^E_u_	207 311 433 529 [221 333 474 571]^34^
Ga_2_O_3_-O1	B_1g_	84 129 145 159 192 211 217 228 257 260 277 291 306 361 393 403 444 460 507 547 615 644 672 695	B_1u_	84 129 145 159 192 211 217 228 257 260 277 291 306 361 393 403 444 460 507 547 615 644 672 695
	B_2g_	64 112 137 158 181 206 239 249 274 296 319 348 357 374 421 473 502 526 581 615 622 639 651 666 727	B_2u_	64 112 137 158 181 206 239 249 274 296 319 348 357 374 421 473 502 526 581 615 622 639 651 666 727
	A_1g_	71 97 116 136 140 161 178 212 247 259 278 300 312 324 347 362 383 427 444 470 513 562 580 605 632 637 696	A_1u_	71 97 116 136 140 161 178 212 247 259 278 300 312 324 347 362 383 427 444 470 513 562 580 605 632 637 696
	A_2g_	71 97 117 142 153 168 171 191 210 230 247 253 275 291 345 367 397 416 434 463 517 552 572 584 642 667 697		
Ga_2_O_3_-O2	A_1g_	100 162 174 260 454 547 604	A_1u_	100 162 174 260 454 547 604
	B_1g_	115 189 227 318 447 560 680	B_1u_	115 189 227 318 447 560 680
	B_2g_	181 273 288 599 637 834	B_2u_	181 273 288 599 637 834
	A_2g_	116 186 211 284 617 640 833		
Ga_2_O_3_-O3	A_g_	65 111 131 173 189 206 220 265 324 419 536 579 593 651 739	B_2u_	88 148 152 187 208 259 263 350 486 564 580 589 631 655
	B_1g_	68 139 179 198 212 267 311 338 372 450 528 558 638 678 777	B_1u_	64 118 187 189 228 244 308 358 444 533 583 607 632 672
	B_3g_	90 123 126 177 194 201 274 302 327 446 564 592 633 689 739	B_3u_	127 154 184 216 245 268 333 366 492 499 545 637 667 681
	B_2g_	115 144 183 192 240 282 304 322 356 453 532 560 641 683 777		

Orthorhombic Ga_2_O_3_-O1, -O2 polytypes are characterized by the *C*_2*v*_(*mm*2) point group, and their phonon modes can be described through the irreducible representation, which encompasses four Raman-active modes (A_1g_, A_2g_, B_1g_, B_2g_) and four infrared-active modes (A_1u_, A_2u_, B_1u_, B_2u_). The most pronounced peaks occur at 697 cm^–1^ (A_2g_) and 454 cm^–1^ (A_1g_) for Ga_2_O_3_-O1 and -O2 polytypes, respectively. The nondegenerate A_1g_ and A_2g_ mode signifies symmetric and antisymmetric stretches or bends with respect to a *C*_2_ axis that is perpendicular to the principal axis, respectively. The infrared absorption peak emerges at 470 and 547 cm^–1^, corresponding to the infrared A_1u_ mode. The orthorhombic Ga_2_O_3_-O3 polytype exhibits symmetry described by the *D*_2*h*_(*mmm*) point group. The corresponding irreducible representation includes four Raman-active modes (A_g_, B_1g_, B_2g_, B_3g_) and three infrared-active modes (B_1u_, B_2u_, B_3u_). The Raman spectroscopy reveals distinct frequency spectra, with the most prominent peak appearing at 593 cm^–1^, corresponding to the Raman-active A_g_ mode. The most significant infrared absorption peak occurs around 444 cm^–1^, correlating with the infrared-active B_1u_ mode. These vibrational modes can be attributed to the bending or stretching vibrations of the oxygen atoms within the plane.

The trigonal Ga_2_O_3_-T polytype exhibits symmetry described by the *D*_*3*d_(*mmm*) point group. The corresponding irreducible representation includes three Raman-active modes (A_1g_, A_2g_, E_g_) and three infrared-active modes (A_1u_, A_2u_, E_u_). Raman spectroscopy shows visible frequency spectra with very few peaks, where the standout peak is situated at 525 cm^–1^, aligning with the Raman-active A_1g_ mode. In an experimental study conducted by Feneberg et al., it was observed that the α-Ga_2_O_3_ crystal structure exhibits Raman-active peaks at 218, 241, 286, 328, 431, 574, and 687 cm^–1^, which closely match the calculated Raman peaks at 201, 223, 265, 309, 403, 525, and 636 cm^–1^.^34^ Additionally, the study revealed IR-active peaks at 221, 271, 333, 474, 546, and 571 cm^–1^, which are found to be near to the calculated IR peaks at 207, 251, 311, 433, 503, and 529 cm^–1^.^34^ The highest absorption peak in the IR spectrum emerges at 433 cm^–1^, corresponding with the infrared-active E_u_ mode. This specific vibrational mode is due to the doubly degenerate asymmetric bending or stretching vibrations of oxygen atoms with respect to the center of symmetry.

### 2.4 Mechanical Stability

Mechanical parameters are calculated for all nine low-energy Ga_2_O_3_ polytypes from elastic tensors. The count of independent components within the stiffness tensor can vary based on the crystal’s symmetry. For monoclinic, trigonal, and orthorhombic phases, the respective numbers are 13, 6, and 9, respectively.^35^ In this study, we determined the second-order elastic constants through the execution of seven discrete ab initio calculations, each involving a distinct level of applied strain ranging from −0.015 to 0.015. The computed second-order elastic constants with existing reported results are presented in Table S3.^36,37^ The elastic constants *C*_11_, *C*_22_, and *C*_33_ are measurements that evaluate the crystal’s resistance to applied mechanical stress along the crystallographic directions *a*, *b*, and *c*, respectively. The value of *C*_33_ is smaller than both *C*_11_ and *C*_22_ in Ga_2_O_3_-M1 and Ga_2_O_3_-M3, suggesting greater compressibility in the *c*-direction. This implies stronger bonding within the ab-plane compared to the out-of-plane directions. In contrast, *C*_22_ exhibits reduced values in other monoclinic polytypes such as Ga_2_O_3_-M2, -M4, and -M5, suggesting increased compressibility in the *b*-direction. In all monoclinic and orthorhombic Ga_2_O_3_ polytypes, *C*_44_ represents the indentation hardness of the crystal, and the little lower values than *C*_66_ observed in all monoclinic polytypes suggest a limited ability to resist shear deformation in the (100) plane. The independent elastic constants from each structure were utilized in the Voigt–Ruess–Hill method to derive various elastic parameters, such as bulk modulus, Young’s modulus, and shear modulus.^38−40^ These results are summarized in Table 3. The eigenvalues of important stiffness tensors for all polytypes are positive. To ensure the stability of the structures, the elastic constants must satisfy the mechanical stability criterion. The Born stability criteria consist of conditions imposed on the elastic constants (*C_ij_*), mainly addressing the second-order alteration in the internal energy of the polytype during deformation.^41^Table S2 provides the Born stability criteria for elastic constants in monoclinic, trigonal, and orthorhombic crystal systems. All individual elastic constants of Ga_2_O_3_ polytypes obey the Born criteria of mechanical stability. This adherence serves as a predictive indicator of the mechanical stability of these polytypes. We employed Hill’s approximation, which closely approximates the mechanical parameters of polycrystalline solids and provides results that align well with the actual conditions.^38^ The bulk modulus values for the Ga_2_O_3_-T polytype, characterized by space group *R*3̅*c*, exceed 200 GPa, greater than those of other polytypes.

**Table 3:** Computed Values of Bulk Modulus *B* (GPa), Shear Modulus *G* (GPa), Poisson’s Ratio ν, Young’s Modulus *E* (GPa), Pugh’s Ratio (*B*/*G*), and Debye Temperature Θ_D_ (K) of All Ga_2_O_3_ Polytypes

polytype name	Ga_2_O_3_-M1	Ga_2_O_3_-M2	Ga_2_O_3_-M3	Ga_2_O_3_-M4	Ga_2_O_3_-M5	Ga_2_O_3_-T	Ga_2_O_3_-O1	Ga_2_O_3_-O2	Ga_2_O_3_-O3
Bulk modulus *B* (GPa)	166.1	157.7 [168.0]^37^	122.9	162.4	85.9	204.1 [217.4]^37^	174.4	181.7	61.6
Shear modulus *G* (GPa)	75.2	69.9 [72.3]^37^	61.5	60.6	40	89.8 [94.8]^37^	66.4	83.1	32.6
Poisson’s ratio (ν)	0.303	0.307 [0.31]^37^	0.286	0.334	0.299	0.308 [0.31]^37^	0.331	0.302	0.275
Young’s modulus *E* (GPa)	195.9	182.8 [189.6]^37^	158	161.7	103.9	234.9 [248.2]^37^	176.7	216.3	83.2
Pugh’s ratio (*B*/*G*)	2.21	2.26	2.00	2.68	2.15	2.27	2.63	2.19	1.89
Debye temperature Θ_D_ (K)	543.2	525.6 [562]^36^	494.8	492.3	404.5	587.1	510.5	567.5	377.2

**Figure 6:**
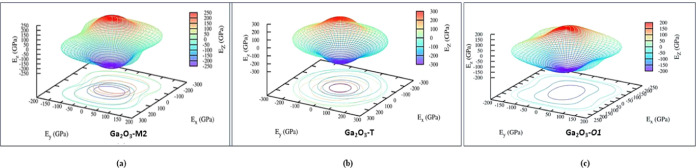
Spatial variation of Young’s modulus for the (a) Ga_2_O_3_-M2, (b) Ga_2_O_3_-T, and (c) Ga_2_O_3_–O1 polytypes, respectively.

Young’s modulus (*E*) measures the toughness of solid compounds and the resistance of a material to longitudinal stress.^42^ Among the Ga_2_O_3_ polytypes, Ga_2_O_3_-T (*E* = 235 GPa) and Ga_2_O_3_-O2 (*E* = 216 GPa) exhibit notably higher values of *E*, indicating a more covalent nature compared to the other polytypes. Conversely, Ga_2_O_3_-O3 displays the lowest *E* value (<100 GPa), signifying its limited ability to withstand significant tensile stress. The Pugh’s ratio (*B*/*G*), where a high value (>1.75) suggests ductility and a low value (≤1.75) implies brittleness, indicates that all stable Ga_2_O_3_ polytypes are expected to demonstrate ductile characteristics since their *B*/*G* values exceed 1.75.^43^ Poisson’s ratio (ν) gauges a crystal’s stability against shear stress and predicts solid failure modes, with a critical value of 0.26. When ν is greater (or lesser) than 0.26, the material is classified as ductile (or brittle).^42,44^ For all mechanically stable Ga_2_O_3_ polytypes, their ν values indicate a ductile nature. The Debye temperature is related to the elastic properties and heat capacity of a solid. The Debye temperature is useful in understanding various properties of solids such as electrical conductivity, thermal conductivity, and thermal expansion. The calculated Θ_D_ values around 500 K shown in Table 3 demonstrate the hardness of all Ga_2_O_3_ polytypes. We present a three-dimensional (3D) spatial representation of Young’s modulus for the Ga_2_O_3_-M2*, -*T, and -O1 polytypes in Figure 6a–c, respectively. The directions *x*, *y*, and *z* correspond to increments along the *a*, *b*, and *c* directions of the primitive cell.

These polytypes display multiple valleys, suggesting inhomogeneity. Refer to Figures S3–S5 in the Supporting Information for Young’s modulus, bulk modulus, and shear anisotropy of other polytypes Ga_2_O_3_-M1, -M3, -M4, -M5, -O2, and -O3 along the *x*, *y*, and *z* directions.

Among the nine low-energy Ga_2_O_3_ polytypes under consideration, our analysis of the mechanical parameters confirms their overall mechanical stability. The investigation into phonon and thermal properties allows us to conclude that except for Ga_2_O_3_-M1, all other polytypes are dynamically stable. Ga_2_O_3_-M1 displays dynamical instability yet remains mechanically stable, leading to its classification as a metastable state. Consequently, all polytypes are considered for further electronic and optical studies.

### 2.5 Electronic Studies

**Figure 7:**
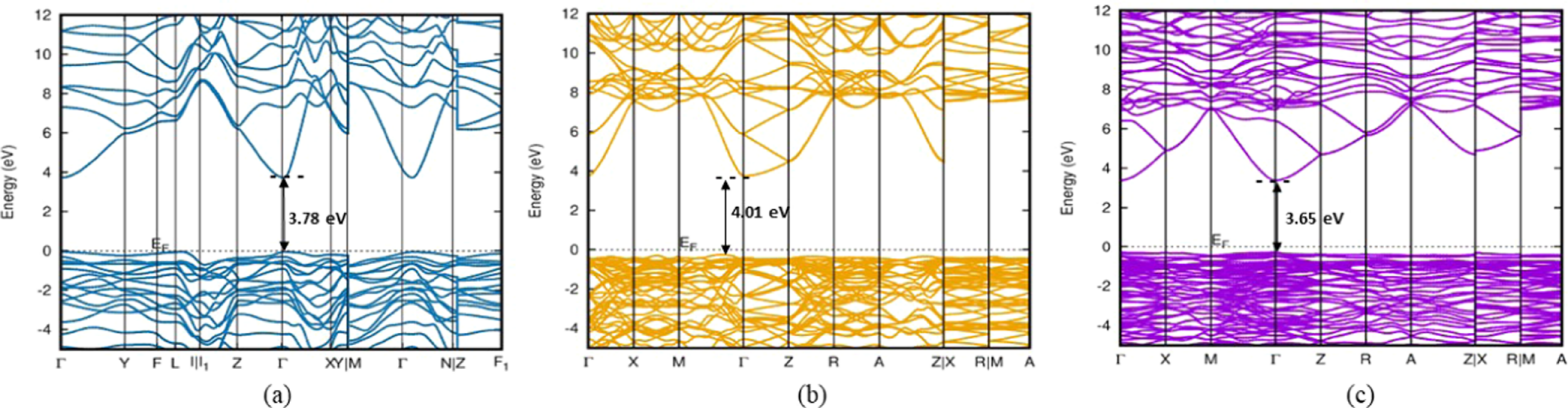
Computed band structures of Ga_2_O_3_ polytypes: (a) Ga_2_O_3_-M2, (b) Ga_2_O_3_-T, and (c) Ga_2_O_3_–O1 using hybrid density functional theory (HSE-06 level).

All polytypes other than Ga_2_O_3_-M1 are shown to be dynamically and mechanically stable through phonon and elastic tensor studies. The practical use of Ga_2_O_3_-like transparent conducting oxides depends on a deep understanding of their unique electronic structure, aside from the need to obtain high-quality single crystals and thin films.^35^ In-depth electronic calculations are carried out to verify and find the viable polytypes for photocatalytic processes, light-emitting diodes, and electronics.^15^ At ambient pressure, we illustrate the band structures and densities of states of all polytypes at the highly symmetric points within the first Brillouin zone. The HSE-06 functional offers a precise representation of the electronic structure in semiconducting materials. Our band structure calculations show that all polytypes exhibit semiconducting behavior (Figures 7 and S6) and the corresponding band gap values are detailed in Table 4.^45^

**Table 4:** Calculated Band Gap Values (HSE-06) of Ga_2_O_3_ Polytypes and Their Corresponding Band Gap Types

polytype name	Ga_2_O_3_-M1	Ga_2_O_3_-M2	Ga_2_O_3_-M3	Ga_2_O_3_-M4	Ga_2_O_3_-M5	Ga_2_O_3_-T	Ga_2_O_3_-O1	Ga_2_O_3_-O2	Ga_2_O_3_-O3
band gap (eV)	1.68	3.78	2.92	3.6	3.29	4.01	3.65	3.84	3.96
		[4.84]^47^				[5.3]^46^	[4.9]^46^		
band gap type	direct	indirect	indirect	indirect	direct	indirect	indirect	direct	indirect

Based on the energy band structures calculated at the HSE-06 level, it is observed that the Ga_2_O_3_-M5 polytype exhibits a relatively lower-energy band gap of 3.29 eV when compared to other polytypes. The polytype Ga_2_O_3_-T possesses the widest band gap, measuring 4.01 eV. The Fermi energy (*E*_F_) is set at the top of the valence bands. The valence band maximum of all Ga_2_O_3_ polytypes lies in the range between −0.34 and 0 eV. The band structure remains consistent across all polytypes, featuring a highly dispersive conduction band minimum at the Γ point and a flat valence band. The band structure of metastable polytype Ga_2_O_3_-M1 is calculated using GGA as it consumes long computational time. Stable monoclinic polytypes, except for Ga_2_O_3_-M5, exhibit indirect semiconductor behavior, with band gap values ranging from 3.29 to 3.78 eV. The calculated band gap for the Ga_2_O_3_-T polytype is 4.01 eV, which is 22% lower than the experimentally confirmed band gap of 5.3 eV for the α-Ga_2_O_3_ polytype.^46^

**Figure 8:**
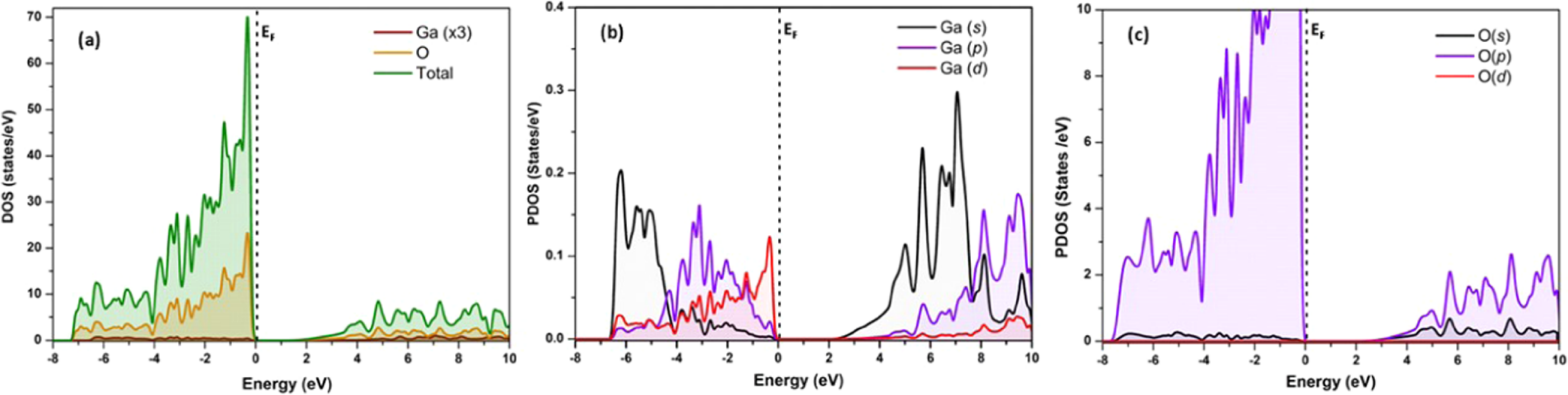
Illustration of calculated (a) total density of states of gallium and oxygen (for better visibility of density of states, Ga contribution has been multiplied by 3 times), (b) projected density of states of gallium orbitals, and (c) projected density of states of oxygen orbitals for Ga_2_O_3_-M3.

Referring to Figure 8a–c our study leads us to conclude that the upper valence bands of Ga_2_O_3_-M3 primarily comprise Ga 3d, 4s, 4p, and O 2p orbitals with a predominant contribution from the O 2p orbital occupies in the energy range from −7.2 to 0 eV. As for the lower conduction bands, they are primarily composed of Ga 4s and O 2p states. The total density of states (DOS) and projected density of states (PDOS) of all other polytypes closely resemble those of the β-Ga_2_O_3_ polytype, consistent with previously reported results.^36^ The calculated density of states for all other stable polytypes is presented in the Supporting Information (Figure S7).

### 2.6 Optical Properties

Ga_2_O_3_ holds a crucial position in the field of optics, owing to its exceptional optical characteristics. These include a high refractive index, a wide optical band gap, minimal optical loss, and exceptional transparency within the visible and near-infrared spectra.^46^ This material finds application in various optical technologies such as high refractive mirrors, versatile broadband interference filters, and active electro-optical devices.^48−50^ Moreover, Ga_2_O_3_ stands out as the preferred choice for antireflection (AR) coatings within the optical industry.^51^ Optical properties such as the dielectric function, absorption, reflectivity, energy loss, and refractive index are determined through the application of GGA and hybrid functional theory, specifically at the HSE-06 level. We employed scissors operator correction for optical spectra.^18^ To accurately compute optical spectra up to photon energies of 30 eV along the parallel direction of polarization (*E*||*X*), it becomes necessary to consider a substantial number of electronic states both above and below the fundamental gap. The key optical properties of a material revolve around its refractive index, denoted as *n*(ω), and its extinction coefficient, represented as *k*(ω). These properties exhibit wavelength dependency and collectively constitute dispersion.^52^ These characteristics are closely related to the complex dielectric function ε(ω). Once we have determined the imaginary component of the dielectric function, it can be employed to derive the real component.^18^ Further, the imaginary part of the dielectric function plays a direct role in the absorption of light.

**Figure 9:**
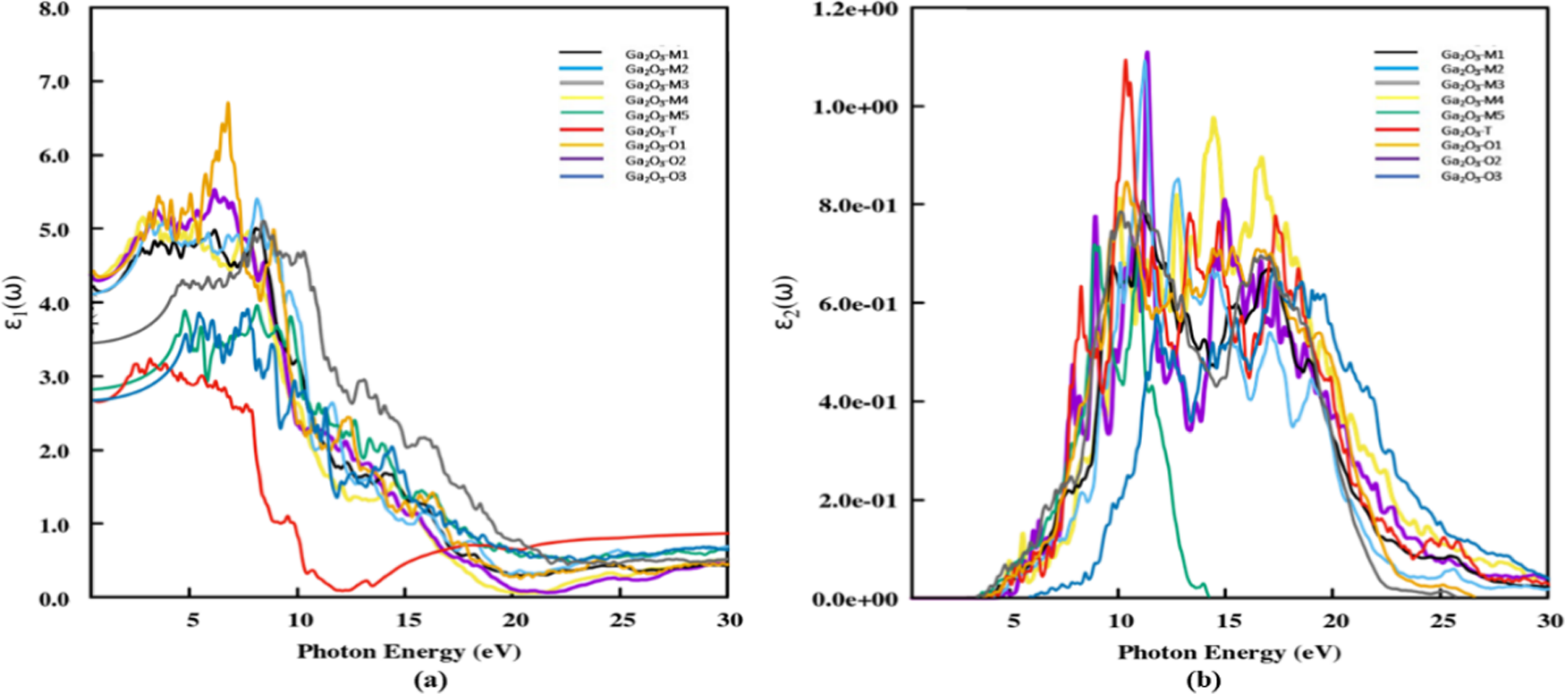
Display of (a) calculated real ε_1_(ω) and (b) imaginary ε_2_(ω) parts of the dielectric functions for photon energy up to 30 eV along the parallel direction of polarization.

The resonant peaks of the polytypes are a result of interband transitions occurring between the valence band maximum (VBM) and conduction band minimum (CBM), as depicted in Figure 9. For all of the Ga_2_O_3_ polytypes, there are several critical points found in the imaginary part and the primary peak extends a wide energy range from 11 to 18 eV. Our calculated results of polytypes Ga_2_O_3_-M2 and Ga_2_O_3_-T are consistent with the results of He et al., from the periodic linear combination of atomic orbitals method for both β-Ga_2_O_3_ and α-Ga_2_O_3_ respectively.^19^

**Figure 10:**
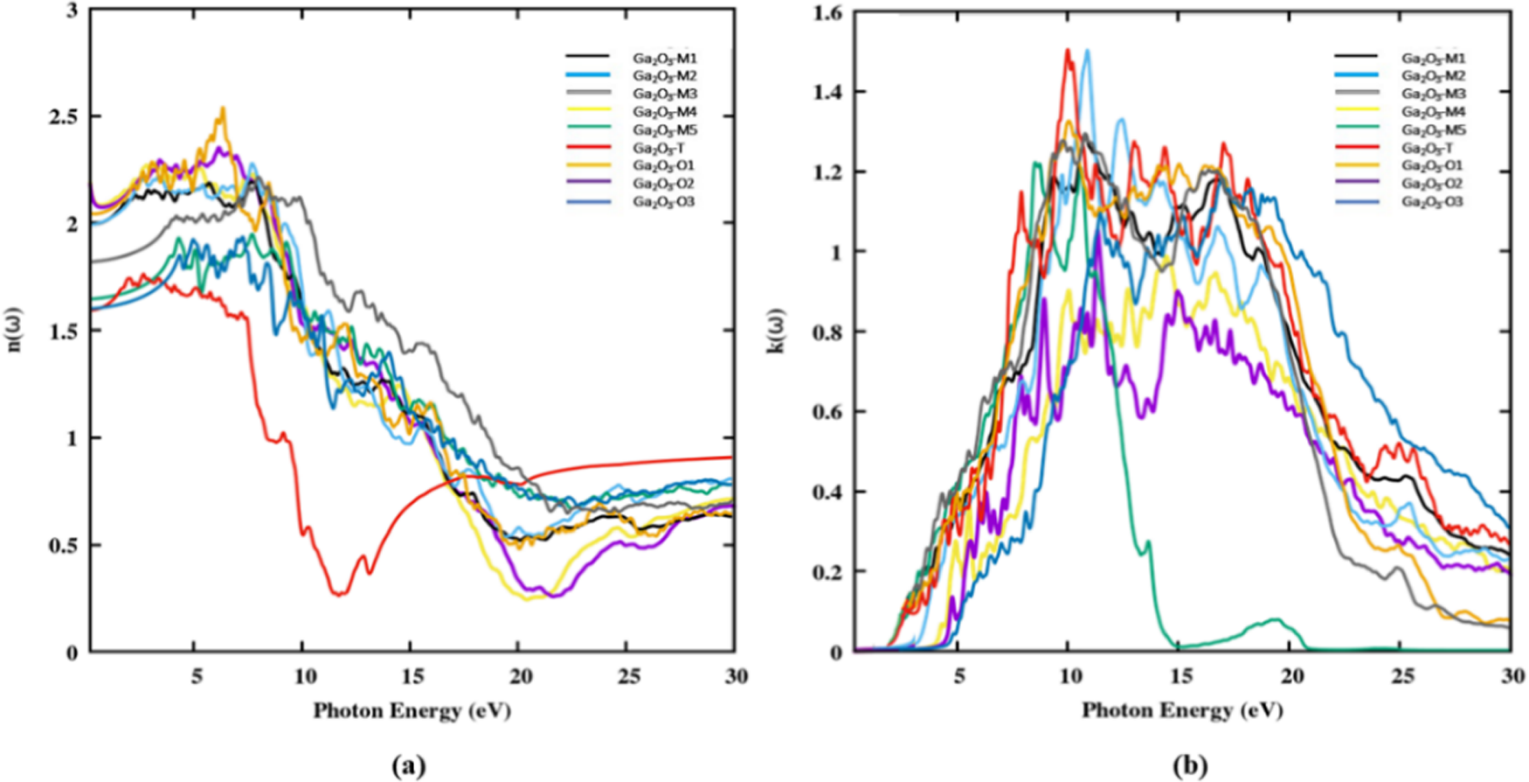
(a) Refractive index *n*(ω) and (b) extinction coefficient *k*(ω) spectra of Ga_2_O_3_ polytypes along the parallel direction of polarization.

The static refractive indices for Ga_2_O_3_ polytypes are presented in Table 5. These polytypes showcase distinct characteristics, featuring broad peaks within the energy range from 4 to 10 eV, corresponding to the points of highest refractive index values. The refractive index exhibits a positive correlation with incident photon frequency, resulting in anomalous dispersion. Similarly, the excitation coefficient *k*(ω) for these polytypes is visually depicted in Figure 10b providing insights into the imaginary component of the dielectric function, ε_2_(ω). Similar to the refractive index *n*(ω), the *k*(ω) spectrum reveals two prominent peaks within the energy range of approximately 9 and 21 eV. Both *n*(ω) and *k*(ω) spectra initially exhibit a rise, indicating the absorption of light by the material in the UV region, followed by a subsequent decline, as illustrated in Figure 10.

**Table 5:** Calculated Optical Dielectric Constant ε_1_(ω), Refractive Index *n*(ω), and Reflectivity *R*(ω) of Ga_2_O_3_ Polytypes with Existing Theoretical and Experimental References

polytype name	Ga_2_O_3_-M1	Ga_2_O_3_-M2	Ga_2_O_3_-M3	Ga_2_O_3_-M4	Ga_2_O_3_-M5	Ga_2_O_3_-T	Ga_2_O_3_-O1	Ga_2_O_3_-O2	Ga_2_O_3_-O3
dielectric constant ε_1_ (ω = 0) *E*||*X*	4.2	4.2 [4.12,^18^ 3.57^55^]	3.4	4.4	2.8	2.6 [3.03,^19^ 3.80^56^]	4.4	4.4	2.6
refractive index *n* (ω = 0)	1.96	1.96 [1.68,^19^ 1.89^55^]	1.75	2.14	1.62	1.55 [1.74,^19^ 1.95^56^]	2.06	2.14	1.55
reflectivity *R* (ω = 0)	0.114	0.114	0.114	0.07	0.06	0.132	0.115	0.07	0.085

**Figure 11:**
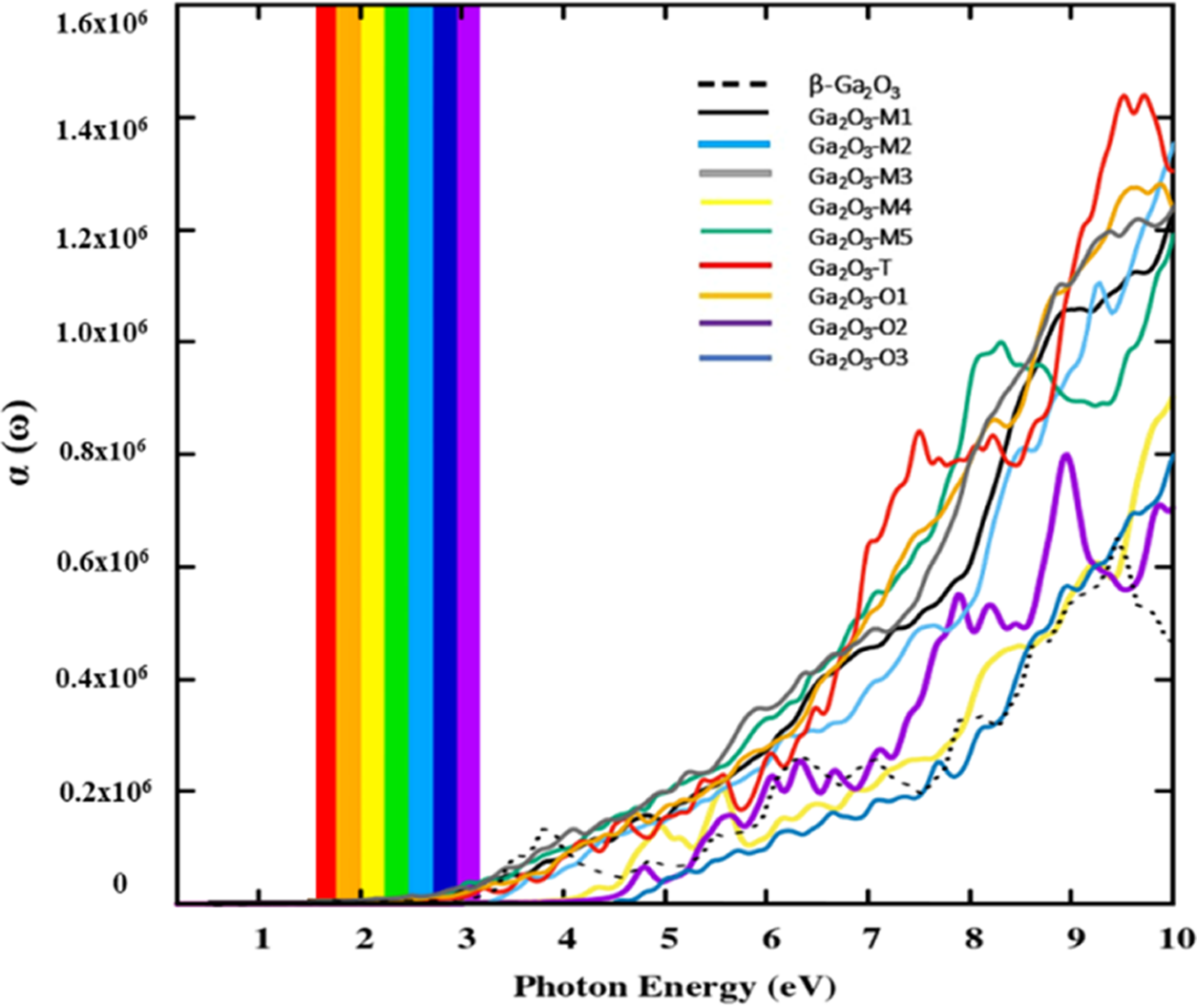
Optical absorption coefficient as a function of photon energy for all Ga_2_O_3_ polytypes with the reference optical absorption spectra of β-Ga_2_O_3_.^53^

The absorption spectra of an optical material are vital because they indicate the amount of light that the material can transmit or absorb. All of the documented polytypes exhibit notable effectiveness in the UV region, with optical absorption observed very low at the visible range, allowing the photon energy to be efficiently transmitted in this energy range, as shown in Figure 11. This makes it an ideal material for solar cell windows. The absorption coefficients of all Ga_2_O_3_ polytypes within the visible range closely match those of the reported β-Ga_2_O_3_ polytype.^45^ In the UV region, the absorption spectra of monoclinic Ga_2_O_3_ polytypes, specifically Ga_2_O_3_-M2 and Ga_2_O_3_-M4, closely resemble the β-Ga_2_O_3_.^53^

**Figure 12:**
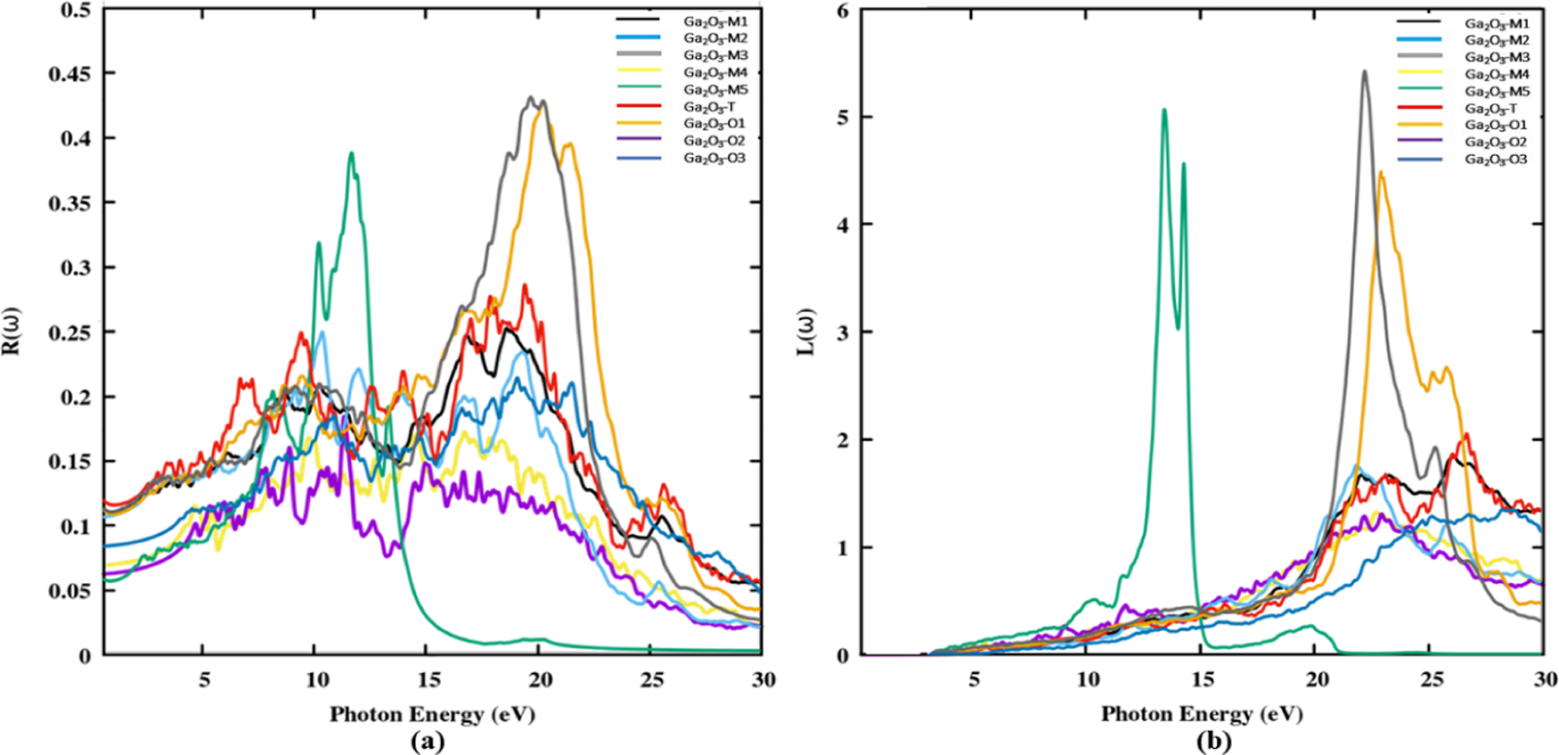
(a) Reflectivity spectra and (b) electron energy loss function (ELF) spectra of Ga_2_O_3_ polytypes along the parallel direction of polarization.

The reflectivity of the Ga_2_O_3_ polytypes is determined by utilizing the calculated extinction coefficient and refractive index. The choice of effective photovoltaic materials is significantly dependent on their optical reflectance properties. Figure 12a showcases the reflectivity characteristics of Ga_2_O_3_ polytypes as a function of the photon energy. Within the visible region, the reflectance of all polytypes falls within the range of 0.06–0.12, with higher values observed in the UV region. The lowest reflectivity is observed in the Ga_2_O_3_-M5 polytype, indicating potential high transmittance or absorption, possibly rendering it more transparent than experimental polytype β-Ga_2_O_3_ at ω = 0. This observation suggests that Ga_2_O_3_-M5 has the lowest reflectivity among all polytypes at zero photon energy. A broad reflection band is evident between 7 and 20 eV for polytypes Ga_2_O_3_-M1, -M2, -M4, -T, -O2, and -O3. These polytypes exhibit a similar pattern of reflectivity, although the intensity may vary within the energy range. However, polytypes exhibit the strongest reflectance at 21, 12, and 21.7 eV for Ga_2_O_3_-M3, -M5, and Ga_2_O_3_-O1, respectively. The reflectivity reaches approximately in the higher-energy region, around 0.43. In the visible spectral ranges, Ga_2_O_3_ polytypes demonstrate less reflectivity, suggesting their potential utility as antireflective or transparent coatings.^54^

Table 5 reveals the optical properties, including dielectric constant, refractive index, and reflectivity at zero frequency. The values are identical for both the metastable polytype Ga_2_O_3_-M1 and the most stable state Ga_2_O_3_-M2, suggesting the potential use of Ga_2_O_3_-M1 in optical devices. Electron energy loss function (ELF), describes the energy dissipated as a fast-moving electron between CBM and VBM. Additionally, the electron’s inelastic scattering behavior is directly associated with the energy loss function. From Figure 12b, we found that there is no observed energy loss below the optical band gap of the polytypes. Instead, the loss becomes prominent during transitions between the valence and conduction bands, steadily increasing until it reaches its peak and subsequently decreases. The energy loss function, *L*(ω), for Ga_2_O_3_-M3, -M5, and Ga_2_O_3_-O1 polytypes approaches its maximum at 22, 12.5, and 23 eV, respectively. Among the 9 polytypes, the Ga_2_O_3_-O3 polytype exhibits the lowest-energy loss function.

## 3 Conclusions

This study focused on the investigation of low-energy Ga_2_O_3_ polytypes for their structural, dynamical, mechanical, electronic, and optical properties under zero-pressure and -temperature conditions. The selection process involved identifying polytypes with low energy levels, resulting in the inclusion of nine polytypes that exhibited energy deviations within 400 meV compared to the lowest-energy structure. The Ga_2_O_3_-M2 system was found to be the most stable, with the Ga_2_O_3_-O1 and Ga_2_O_3_-T systems being energetically close. The phonon calculations conducted for the Ga_2_O_3_ polytypes using VASP allowed for a comprehensive understanding of their dynamic and thermal properties. Phonon calculations indicated that all polytypes except Ga_2_O_3_-M1 were dynamically stable. In dynamically stable polytypes, acoustic frequencies are governed by the heavier gallium atoms, while optical frequencies are primarily influenced by the smaller oxygen atoms. The polytypes Ga_2_O_3_-O2 and Ga_2_O_3_-O3 exhibit a distinct phonon band gap between optical and acoustic modes, influencing the phonon scattering mechanisms and thermal conductivity.

All stable polytypes exhibit a higher heat capacity, ensuring enhanced stability even at elevated temperatures. Raman and infrared (IR) spectroscopies were employed to characterize the phonon properties of the polytypes. Further, the mechanical stability assessment involved calculating second-order elastic constants for all nine low-energy polytypes using elastic tensors. It was determined that the Ga_2_O_3_-M1 polytype exhibits metastable, while all other polytypes are found to be dynamically and mechanically stable through phonon and elastic tensor studies. Ga_2_O_3_ polytypes exhibit different Young’s modulus values, indicating variations in covalent nature and ability to withstand tensile stress. All Ga_2_O_3_ polytypes are expected to demonstrate ductile characteristics based on their Pugh’s ratio values. Poisson’s ratio indicates the stability against shear stress and solid failure modes. Dynamical and mechanical analyses further narrowed down the viable polytypes for electronic and optical studies. Electronic calculations using the HSE-06 functional provided accurate descriptions of the band structures and density of states for these stable polytypes. These findings from this investigation contribute to a better understanding of Ga_2_O_3_ polytypes, facilitating their potential applications in various fields such as optoelectronics and electronics. The derived information on the refractive index, extinction coefficient, and dielectric function provides a foundation for further understanding of the optical properties of Ga_2_O_3_ polytypes and their applications in various fields such as photocatalysis, light-emitting diodes, and electronics. Ga_2_O_3_-M3, -M5, and Ga_2_O_3_-O1 polytypes exhibit strong reflectance at specific energy levels, making them potential candidates for coating materials. Further experimental investigations can build upon these findings to validate and expand our understanding of Ga_2_O_3_ polytypes and their properties.

## 4 Computational Details

Employing density functional theory, we conducted geometric optimization and electronic structure calculations for various Ga_2_O_3_ polytypes using the VASP.^57^ The interaction between core and valence electrons of gallium and oxygen (Ga: [Ar] 3d^10^4s^2^4p^1^, O: 1s^2^2s^2^2p^4^) is treated through the projector augmented-wave (PAW) method.^58^ The Perdew, Burke, and Ernzerhof (PBE) parametrized generalized gradient approximation (GGA) is employed for exchange-correlation functional, augmented with a self-interaction correction scheme.^26^ The optimized crystal structure, along with lattice parameters, site coordinates, and Wyckoff positions, is analyzed using VESTA, a tool for three-dimensional visualization.^59^ Monkhorst–Pack grid scheme is employed for *k*-point sampling within the Brillouin zone for the polytypes.^60^ Optimization reaches convergence with an energy cutoff value of 550 eV. Phonon calculations employ the supercell approach, with the PHONOPY code determining phonon frequencies and density of states.^27^ Raman and IR spectra for all polytypes are acquired using density functional perturbation theory, as implemented in the CASTEP package.^61^ Elastic constants are computed using the finite strain method with the VASPKIT post-pre-processing tool.^62^ Band structures and optical spectra are obtained via the Heyd–Scuseria–Ernzerhof (HSE-06) screened hybrid functional.^63^ To accurately compute optical spectra up to photon energies of 30 eV, we incorporated a significant number of electronic states and employed a higher *k*-point grid, which enhances the smoothness of the dielectric function.

## Supporting Information Available

Structural information on Ga_2_O_3_ polytypes; Raman- and IR-active modes; phonon dispersions; spatial-dependent mechanical properties of the Ga_2_O_3_ polytypes, and electronic band structures (PDF)
