# Supplementary material for: Investigating Stable Low-Energy Gallium Oxide (Ga2O3) Polytypes: Insights into Electronic and Optical Properties from First Principles
Source: ACS Omega. 2024 Mar 26;9(14):16207–20. doi: 10.1021/acsomega.3c10192 (PMC11007711; doi:10.1021/acsomega.3c10192)
Supplement: Supplementary file 1 — ao3c10192_si_001.pdf [file ao3c10192_si_001.pdf]

Supplementary information of "Investigating stable low-energy gallium oxide (Ga_2O_3) polytypes: Insights into electronic and optical properties from first-principles"

Arthi Devamanoharan [1], Vishnukanthan Venkatachalapathy[2,3], Vasu Veerapandy[1] and Ponniah Vajeeston[4*]

[1]*School of Physics, Madurai Kamaraj University, Madurai 625021, India.*

[2]*Department of Physics/Centre for Materials Science and Nanotechnology, University of Oslo, P.O Box 1048 Blindern, NO-0316 Oslo, Norway*

[3]*Department of Materials Science, National Research Nuclear University "MEPhI", 31 Kashirskoe sh, Moscow, Russian Federation*

[4]*Center for Materials Science and Nanotechnology, University of Oslo, Oslo 0371, Norway.*

1. Structural information of Ga_2O_3 polytypes

Ga_2O_3-*M1* is beta indium sulfide-like structured and crystallizes in the monoclinic *C2/c* space group. There are five inequivalent Ga sites. In the first Ga site, Ga(1) is bonded to six O atoms to form GaO_6 octahedra. In the second Ga site, Ga(2) is also bonded to six O atoms to form GaO_6 octahedra. In the third Ga site, Ga(3) is bonded to four O atoms to form corner-sharing GaO_4 tetrahedra. In the fourth Ga site, Ga(4) is bonded to six O atoms to form GaO_6 octahedra. In the fifth Ga site, Ga(5) is bonded to four O atoms to form corner-sharing GaO_4 tetrahedra. There are six inequivalent O sites. In the first O site, O(1) is bonded in a distorted trigonal planar geometry to three Ga atoms. In the second O site, O(2) is bonded in a distorted trigonal planar geometry to three Ga atoms. In the third O site, O(3) is bonded in a distorted trigonal pyramidal geometry to four Ga atoms. In the fourth O site, O(4) is bonded in a distorted trigonal planar geometry to three Ga atom. In the fifth O site, O(5) is bonded in a rectangular see-saw-like geometry to four Ga atoms. In the sixth O site, O(6) is bonded in a distorted rectangular see-saw-like geometry to four Ga atoms.

Ga_2O_3-*M2* crystallizes within the monoclinic *C2/m* space group. The crystal structure features two distinct Ga sites. In the initial Ga site, Ga(1) forms GaO_4 tetrahedra through bonds with one O(1), one O(3), and two equivalent O(2) atoms. In the subsequent Ga site, Ga(2) creates GaO_6 octahedra by interacting with one O(2), two equivalent O(1), and three equivalent O(3) atoms. There are three distinct O sites. The first O site, O(1), adopts a trigonal planar arrangement and forms bonds with one Ga(1) and two equivalent Ga(2) atoms. Similarly, the second O site, O(2), exhibits a trigonal planar coordination with one Ga(2) and

* Corresponding author

E-mail address: vajeeston.ponniah@kjemi.uio.no ; https://folk.universitetetioslo.no/ponniahv/

two equivalent Ga(1) atoms. Lastly, the third O site, O(3), assumes a distorted tetrahedral configuration, bonding with one Ga(1) and three equivalent Ga(2) atoms.

Ga_2O_3-*M4* crystallizes in the monoclinic $P2_1/c$ space group. There are two inequivalent Ga sites. In the first Ga site, Ga(1) is bonded to one O(3), two equivalent O(1), and two equivalent O(2) atoms to form GaO_5 trigonal bipyramids. In the second Ga site, Ga(2) is bonded to one O(1), one O(2), and two equivalent O(3) atoms to form GaO_4 tetrahedra. There are three inequivalent O sites. In the first O site, O(1) is bonded in a distorted trigonal planar geometry to one Ga(2) and two equivalent Ga(1) atoms. In the second O site, O(2) is bonded in a distorted T-shaped geometry to one Ga(2) and two equivalent Ga(1) atoms. In the third O site, O(3) is bonded in a distorted trigonal planar geometry to one Ga(1) and two equivalent Ga(2) atoms.

Ga_2O_3-*M5* crystallizes in the monoclinic $P2_1/c$ space group. There are two inequivalent Ga sites. In the first Ga site, Ga(1) is bonded to one O(1), one O(3), and three equivalent O(2) atoms to form a mixture of distorted corner and edge-sharing GaO_5 trigonal bipyramids. In the second Ga site, Ga(2) is bonded to one O(2), two equivalent O(1), and two equivalent O(3) atoms to form a mixture of corner and edge-sharing GaO_5 trigonal bipyramids. There are three inequivalent O sites. In the first O site, O(1) is bonded in a trigonal planar geometry to one Ga(1) and two equivalent Ga(2) atoms. In the second O site, O(2) is bonded in a distorted trigonal pyramidal geometry to one Ga(2) and three equivalent Ga(1) atoms. In the third O site, O(3) is bonded in a trigonal planar geometry to one Ga(1) and two equivalent Ga(2) atoms.

Ga_2O_3-*O1* crystallizes in the orthorhombic $Pna2_1$ space group. There are four inequivalent Ga sites. In the first Ga site, Ga(1) is bonded to six O atoms to form distorted GaO_6 octahedra. In the second Ga site, Ga(2) is bonded to one O(3), one O(4) and two equivalent O(1) atoms to form GaO_4 tetrahedra. In the third Ga site, Ga(3) is bonded to one O(3), one O(6), two equivalent O(2), and two equivalent O(4) atoms to form GaO_6 octahedra that share corners with three equivalent Ga(2)O_4 tetrahedra. In the fourth Ga site, Ga(4) is bonded in a 6-coordinate geometry to one O(3), one O(6), two equivalent O(2), and two equivalent O(5) atoms. There are six inequivalent O sites. In the first O site, O(5) is bonded in a trigonal planar geometry to one $Ga(1)^{3+}$ and two equivalent Ga(4) atoms. In the second O site, O(6) is bonded in a trigonal planar geometry to one Ga(1), one Ga(3), and one Ga(4) atom. In the third O site, O(1) is bonded in a trigonal planar geometry to one Ga(1) and two equivalent Ga(2) atoms. In the fourth O site, O(2) is bonded in a distorted square pyramidal geometry to one Ga(1), two equivalent Ga(3), and two equivalent Ga(4) atoms. In the fifth O site, O(3) is bonded in a tetrahedral geometry to one Ga(1), one Ga(2), one Ga(3), and one Ga(4) atom. In the sixth O site, O(4) is bonded in a distorted tetrahedral geometry to one Ga(1), one Ga(2), and two equivalent Ga(3) atoms.

2. Wyckoff positional parameters of Ga_2O_3 polytypes

Table S1: The optimized equilibrium atomic positional parameters with corresponding sites of Ga_2O_3 polytypes

Polytype Name	Atom	Site	Coordinates

Space Group			x	y	z
Ga_2O_3-*M1*	Ga(1)	8*f*	0.091	0.26853	0.1589
	Ga(2)	8*f*	0.16223	0.47717	0.84514
	Ga(3)	8*f*	0.17802	0.35715	0.58333
	Ga(4)	4*a*	0	0	0
	Ga(5)	4*e*	0	0.11616	0.75000
	O(1)	8*f*	0.00272	0.23811	0.49613
	O(2)	8*f*	0.08067	0.50663	0.17007
	O(3)	8*f*	0.0933	0.01997	0.16377
	O(4)	8*f*	0.14838	0.27475	0.32468
	O(5)	8*f*	0.17317	0.24915	0.82656
	O(6)	8*f*	0.23842	0.00537	0.50145
Ga_2O_3-*M2*	Ga(1)	4*i*	0.90962	0	0.20479
	Ga(2)	4*i*	0.65839	0	0.31421
	O(1)	4*i*	0.83576	0	0.89048
	O(2)	4*i*	0.50413	0	0.74408
	O(3)	4*i*	0.1735	0	0.56363
Ga_2O_3-*M3*	Ga(1)	2*a*	0.22827	0	0.38524
	Ga(2)	4*b*	0.48725	0.25984	0.50414
	Ga(3)	2*a*	0.07823	0	0.96817
	Ga(4)	4*b*	0.31571	0.25231	0.8353
	Ga(5)	4*b*	0.15368	0.23531	0.17512
	Ga(6)	2*a*	0.40705	0.25000	0.29032
	Ga(7)	2*a*	0.05281	0	0.70483
	Ga(8)	2*a*	0.25192	0.25000	0.01481
	Ga(9)	2*a*	0.05708	0.25000	0.33333
	Ga(10)	2*a*	0.06808	0.25000	0.83478
	Ga(11)	2*a*	0.40273	0	0.0562
	Ga(12)	2*a*	0.23344	0.25000	0.52855
	Ga(13)	2*a*	0.37506	0	0.65455
	O(1)	4*b*	0.47361	0.23346	0.75584
	O(2)	2*a*	0.23001	0	0.25551
	O(3)	2*a*	0.07294	0	0.0948
	O(4)	4*b*	0.33533	0.25848	0.58218
	O(5)	2*a*	0.07399	0	0.57946
	O(6)	4*b*	0.30698	0.2506	0.08908
	O(7)	2*a*	0.39783	0.25000	0.92163
	O(8)	4*b*	0.15868	0.26307	0.91875
	O(9)	2*a*	0.40287	0.25000	0.4255
	O(10)	4*b*	0.1464	0.27205	0.42794
	O(11)	4*b*	0.49847	0.75611	0.25399
	O(12)	2*a*	0.22485	0.25000	0.25221
	O(13)	2*a*	0.23155	0.25000	0.77186
	O(14)	2*a*	0.0837	0.25000	0.08906
	O(15)	2*a*	0.05701	0.25000	0.58036
	O(16)	2*a*	0.40997	0	0.42117
	O(17)	2*a*	0.40255	0	0.91123
	O(18)	2*a*	0.23775	0	0.75312
Ga_2O_3-*M4*	Ga(1)	4*e*	0.94004	0.54192	0.72709
	Ga(2)	4*e*	0.52377	0.65495	0.20721
	O(1)	4*e*	0.83679	0.11584	0.39814
	O(2)	4*e*	0.74633	0.02518	0.81403

	O(3)	4*e*	0.67232	0.70332	0.57309
Ga_2O_3-*M5*	Ga(1)	4*e*	0.83568	0.06517	0.69938
	Ga(2)	4*e*	0.60082	0.16672	0.17953
	O(1)	4*e*	0.96418	0.62454	0.4693
	O(2)	4*e*	0.66929	0.49299	0.80883
	O(3)	4*e*	0.63147	0.19413	0.5264
Ga_2O_3-*T*	Ga(1)	4*c*	0.85518	0.85518	0.85518
	O(1)	6*e*	0.55348	0.94652	0.25
Ga_2O_3-*O1*	Ga(1)	4*a*	0.18054	0.03276	0.00381
	Ga(2)	4*a*	0.18047	0.34839	0.20569
	Ga(3)	4*a*	0.18714	0.66175	0.01708
	Ga(4)	4*a*	0.19397	0.34929	0.79591
	O(1)	4*a*	0.02391	0.16777	0.14695
	O(2)	4*a*	0.02845	0.82399	0.13557
	O(3)	4*a*	0.0221	0.48854	0.64191
	O(4)	4*a*	0.1543	0.34201	0.40595
	O(5)	4*a*	0.1476	0.67228	0.38065
	O(6)	4*a*	0.14911	0.00223	0.41259
	Ga(1)	8*b*	0.83981	0.17674	0.42700
Ga_2O_3-*O2*	O(1)	8*b*	0.79712	0.13103	0.07037
	O(2)	4*a*	0	0.27121	0.53300
	Ga(1)	8*c*	0.44347	0.80418	0.12695
	Ga(2)	8*c*	0.65811	0.43146	0.90321
Ga_2O_3-*O3*	O(1)	8*c*	0.08602	0.19187	0.27497
	O(2)	8*c*	0.63538	0.53065	0.1617
	O(3)	8*c*	0.71912	0.86088	0.18094

3. Dynamical stability

Figure S1 represents the phonon dispersion with PhDOS of remaining Ga_2O_3 polytypes.

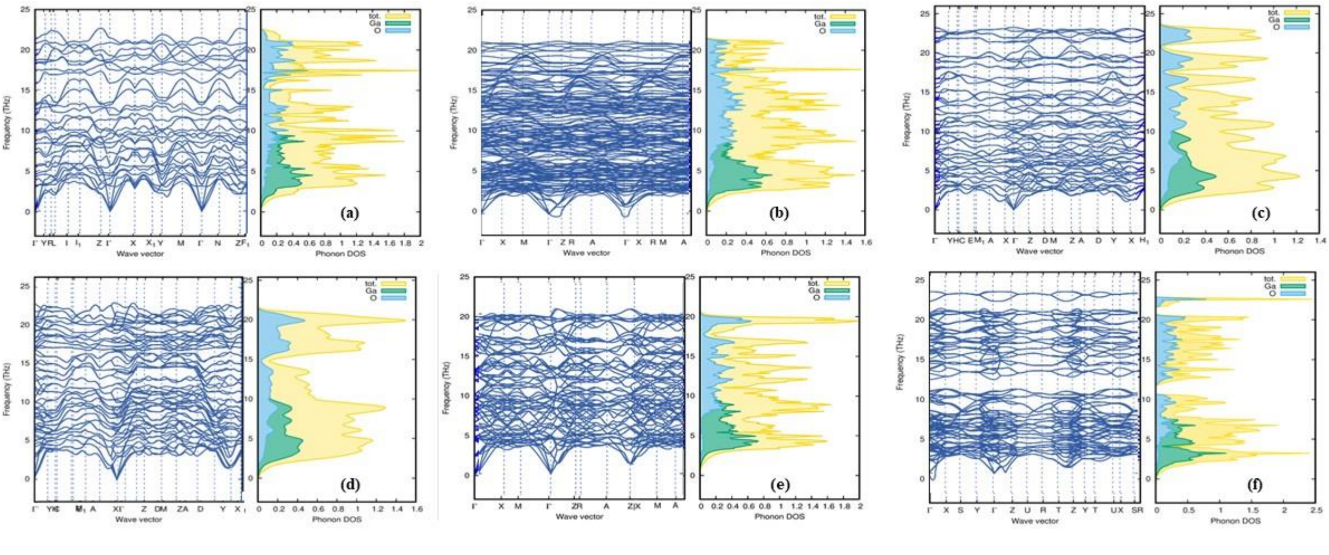

Figure S1: Phonon dispersion curves with PhDOS of Ga_2O_3 polytypes: (a) Ga_2O_3-*M2* (b) Ga_2O_3-*M3* (c) Ga_2O_3-*M4* (d) Ga_2O_3-*M5* (e) Ga_2O_3-*O2* and (f) Ga_2O_3-*O3*

4. Raman and IR active modes

Figure S2 represents Raman –IR spectra of Ga_2O_3 polytypes.

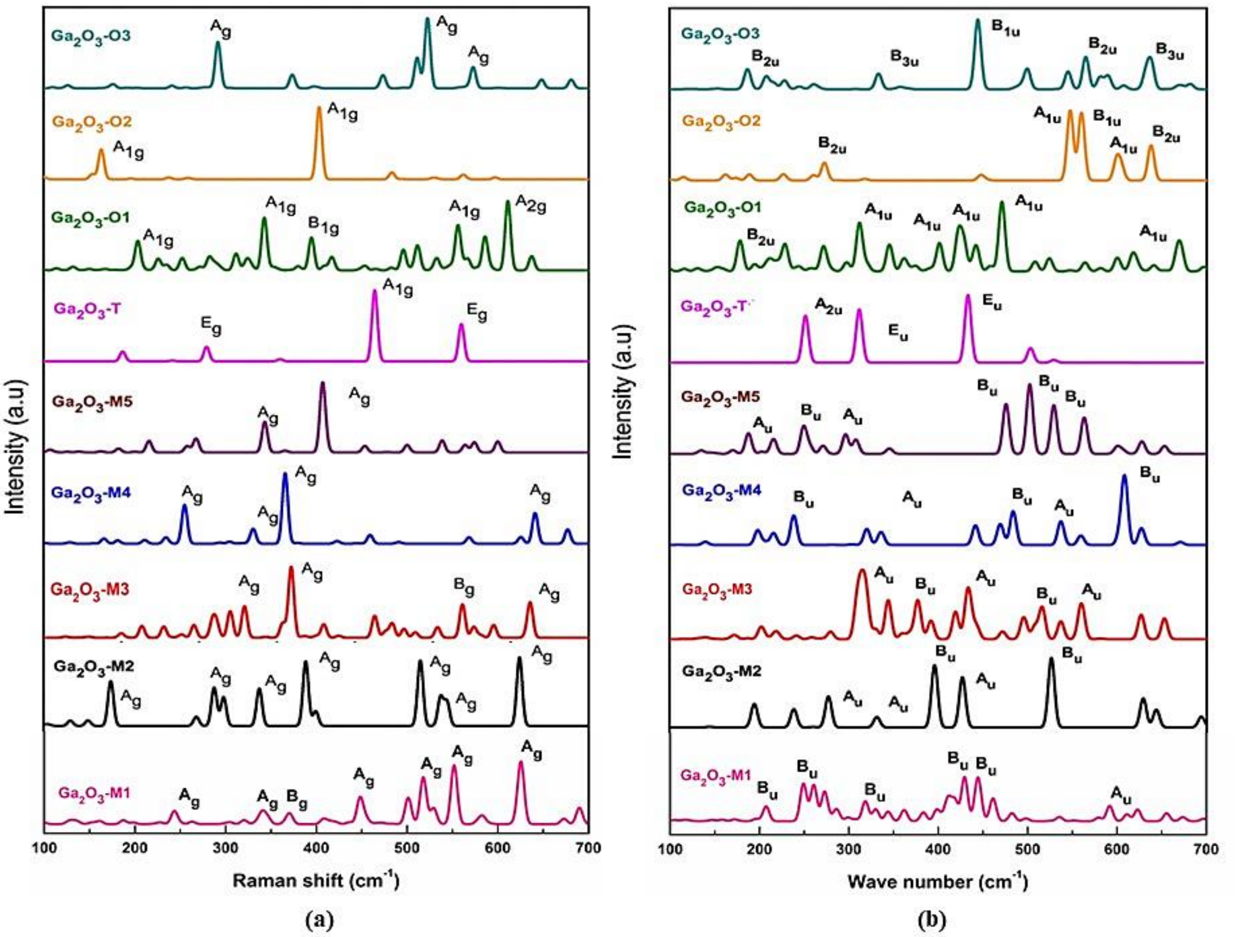

Figure S2: The (a) Raman and (b) IR spectra of Ga_2O_3 polytypes

5. Mechanical properties

Table S2 provides Born stability criteria of different crystal systems and Table S3 contains computed value of elastic constants C_{ij} (GPa) of all Ga_2O_3 polytypes

Table S2: Crystal systems with their corresponding Born stability criteria

Crystal System	Born stability criteria
Monoclinic	$C_{11}, C_{22}, C_{33}, C_{44}, C_{55}, C_{66} > 0$; $[C_{11} + C_{22} + C_{33} + 2(C_{12} + C_{13} + C_{23})] > 0$; $C_{33}C_{55} - C_{35}^2 > 0$; $C_{44}C_{66} - C_{46}^2 > 0$; $C_{22} + C_{33} - 2C_{23} > 0$
Trigonal	$C_{11} - C_{12} > 0$; $C_{13}^2 < 0.5\, C_{33}(C_{11} + C_{12})$; $C_{14}^2 < 0.5\, C_{44}(C_{11} - C_{12})$; $C_{44} > 0$
Orthorhombic	$C_{11}, C_{44}, C_{55}, C_{66} > 0$; $C_{11}C_{22} > C_{12}^2$; $C_{11}C_{22}C_{33} + 2C_{12}C_{13}C_{23} - C_{11}C_{23}^2 - C_{22}C_{13}^2 - C_{33}C_{12}^2 > 0$

Table S3: The computed values of single crystal elastic constants C_{ij} (GPa) of all Ga_2O_3 polytypes

Polytype Name	Ga_2O_3-*M1*	Ga_2O_3-*M2*	Ga_2O_3-*M3*	Ga_2O_3-*M4*	Ga_2O_3-*M5*	Ga_2O_3-*T*	Ga_2O_3-*O1*	Ga_2O_3-*O2*	Ga_2O_3-*O3*
C_{11}	273.6	313.7 [242.8][37]	261.1	254.8	184.8	359.4 [380.3][38]	300.5	317.4	161.9
C_{12}	139.9	109.8 [128.0][37]	93.3	132.4	59.6	163.2 [174.0][38]	147.6	112.1	45.8

***C*13**	114.3	68.6 [160.0][37]	63.7	116.1	62.7	117.5 [128.5][38]	119.3	111.7	35.6
***C*15**	34.9	0 [-1.6][37]	-0.26	14.8	-5.9	16.7 [-16.6][38]			
***C*22**	235.6	211.5 [343.8][37]	239.6	211.7	84.3		252.7	322.9	88.2
***C*23**	134.9	117.6 [70.9][37]	54.7	117.5	68.9		115.9	113.9	20.7
***C*25**	2.34	17 [0.4][37]	0.8	-17.9	-10.7				
***C*33**	216.4	312.2 [347.4][37]	204.4	268.9	208.6	329.9 [342.9][38]	259.9	319.8	124.9
***C*35**	-19.5	-6.19 [1.0][37]	-0.098	5.6	-16.4				
***C*44**	97.5	64.7 [47.8][37]	58.2	60.9	41.8	74.5 [80.1][38]	68.8	71.7	42.9
***C*46**	2.79	-16.2 [5.6][37]	0.16	-10.9	-17.4				
***C*55**	83.3	46.1 [88.6][37]	45.5	71.3	38.8		43.3	71.7	17.4
***C*66**	114.8	88.9 [104.0][37]	78.1	59.3	44.8	98.1	86.3	71.4	25.8

Figures S3, S4 and S5 provide 3D spatial representations for Young's modulus, bulk modulus, and shear modulus of remaining Ga_2O_3 polytypes.

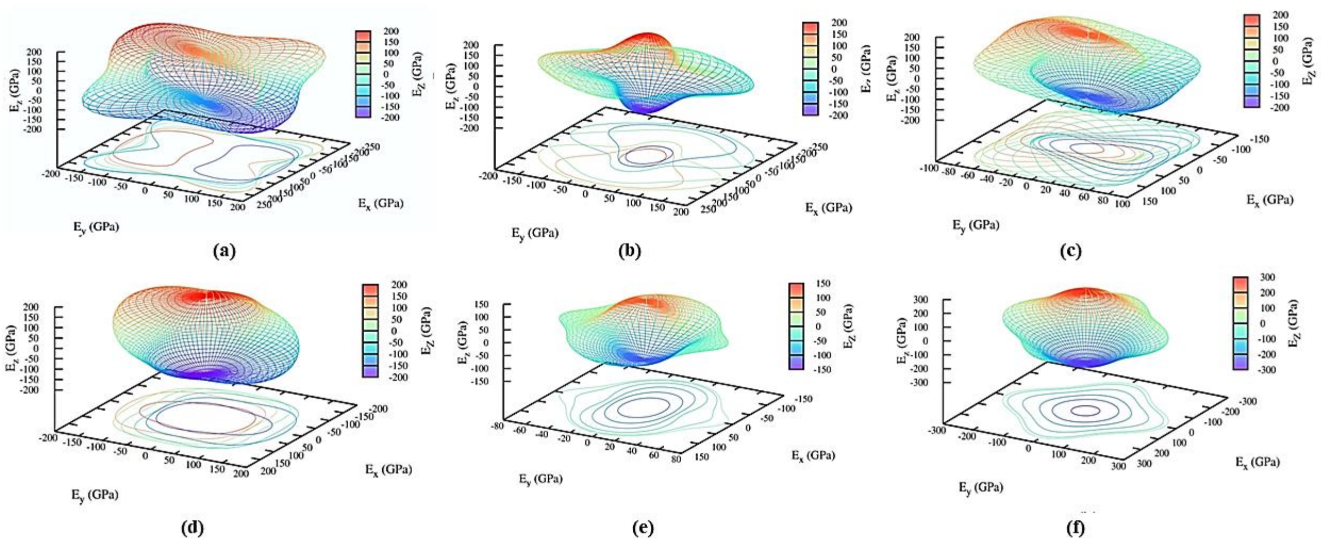

Figure S3: Young's modulus of Ga_2O_3 polytypes (a) Ga_2O_3-*M1* (b) Ga_2O_3-*M3* (c) Ga_2O_3-*M4* (d) Ga_2O_3-*M5* (e) Ga_2O_3-*O2* and (f) Ga_2O_3-*O3*

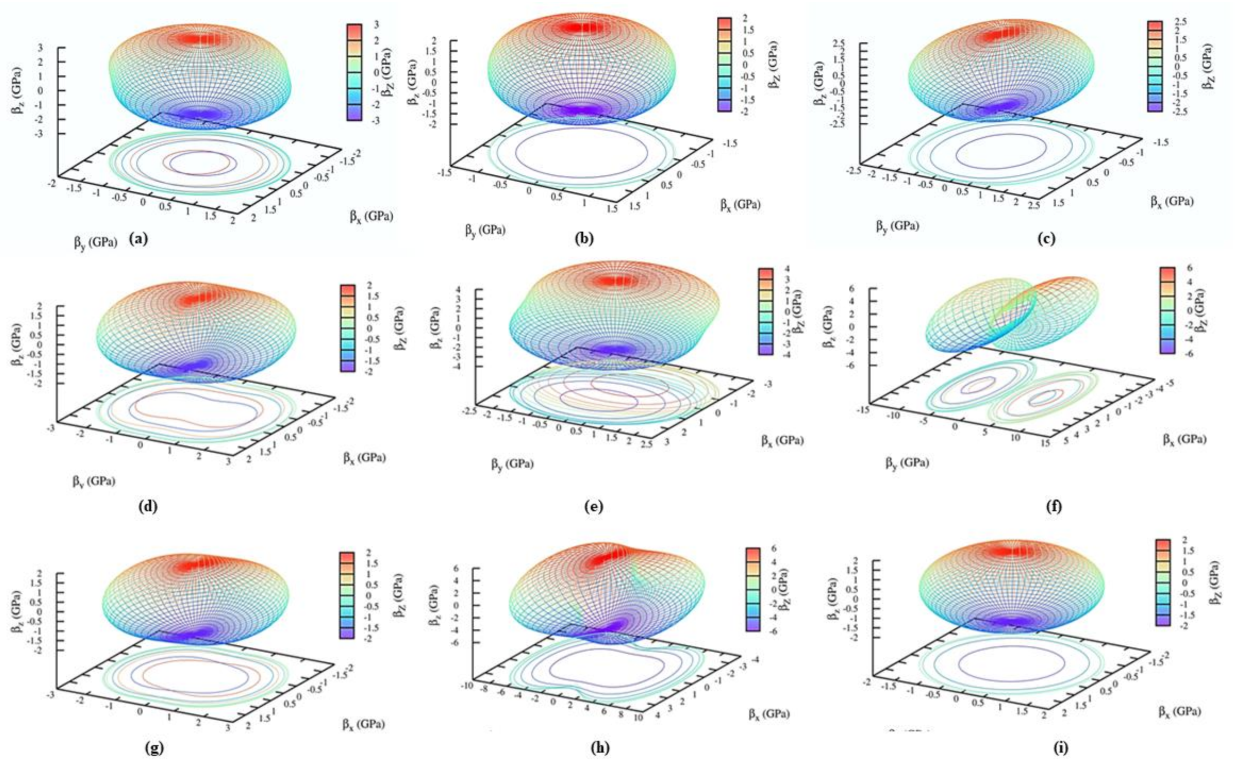

Figure S4: Bulk modulus of Ga_2O_3 polytypes (a) Ga_2O_3-*M1* (b) Ga_2O_3-*T* (c) Ga_2O_3-O*1* (d) Ga_2O_3-*M2* (e) Ga_2O_3-*M3* (f) Ga_2O_3-*M4* (g) Ga_2O_3-*M5* (h) Ga_2O_3-*O2* and (i) Ga_2O_3-*O3*

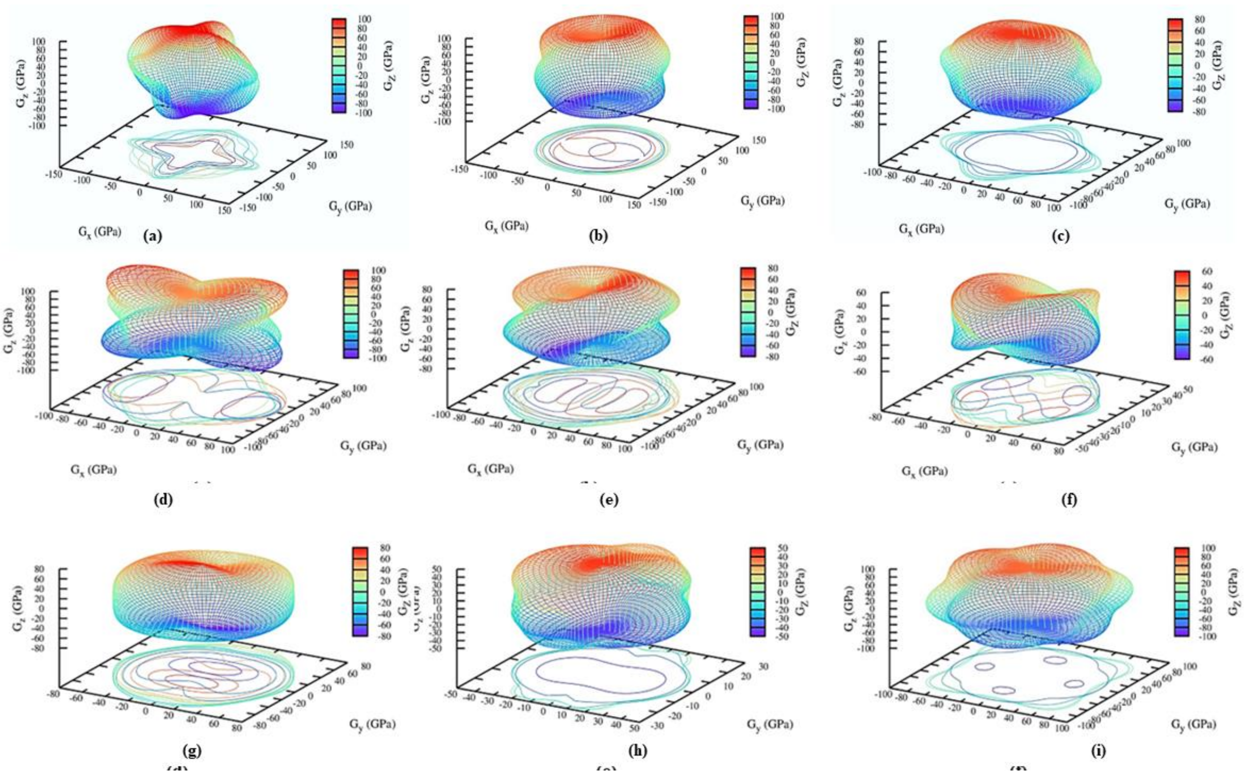

Figure S5: Shear modulus of Ga_2O_3 polytypes (a) Ga_2O_3-*M1* (b) Ga_2O_3-*T* (c) Ga_2O_3-O*1* (d) Ga_2O_3-*M2* (e) Ga_2O_3-*M3* (f) Ga_2O_3-*M4* (g) Ga_2O_3-*M5* (h) Ga_2O_3-*O2* and (i) Ga_2O_3-*O3*

6. Electronic studies

Figure S6 represents electronic band with their corresponding band gap value of remaining Ga_2O_3 polytypes and Figure S7 exhibits the total density of states of Ga_2O_3 polytypes.

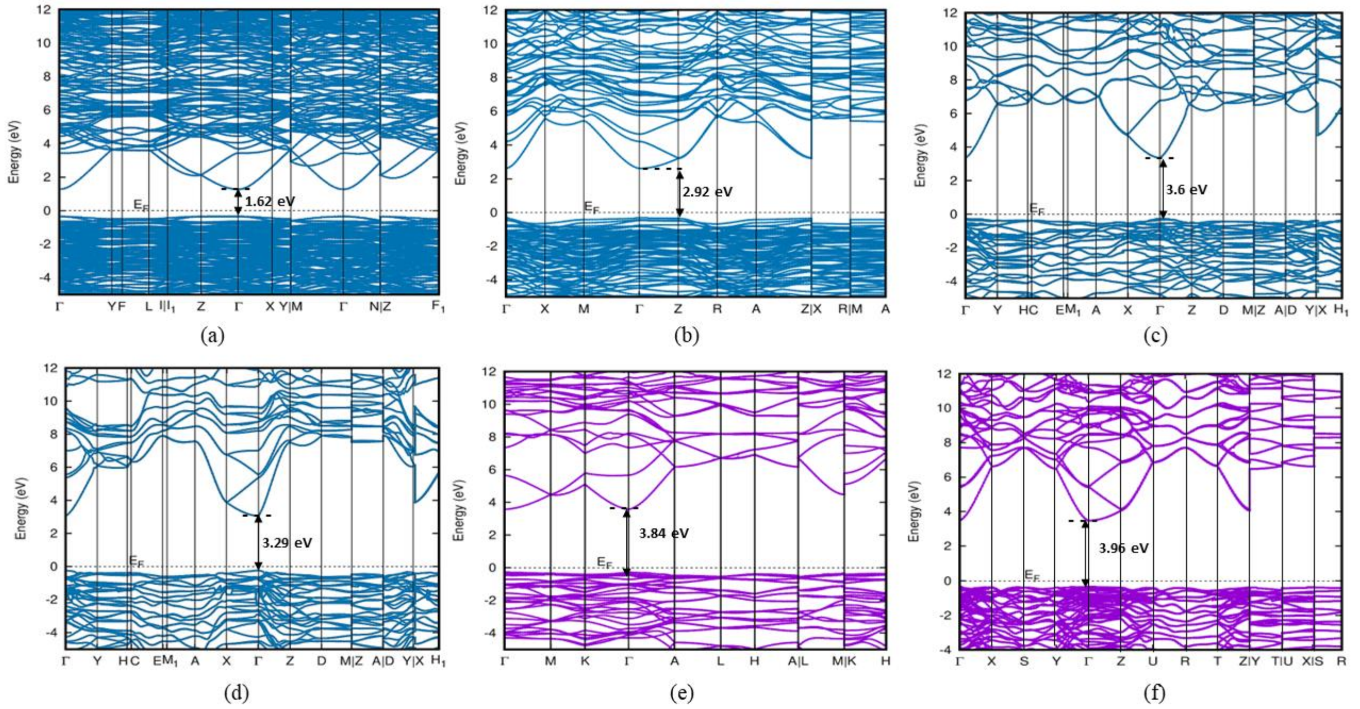

Figure S6: Computed band structures Ga_2O_3 polytypes (a) Ga_2O_3-*M2* (calculated using *GGA)* (b) Ga_2O_3-*M3* (c) Ga_2O_3-*M4* (d) Ga_2O_3-*M5* (e) Ga_2O_3-*O2* and (f) Ga_2O_3-*O3* calculated using hybrid density functional theory (HSE-06 level)

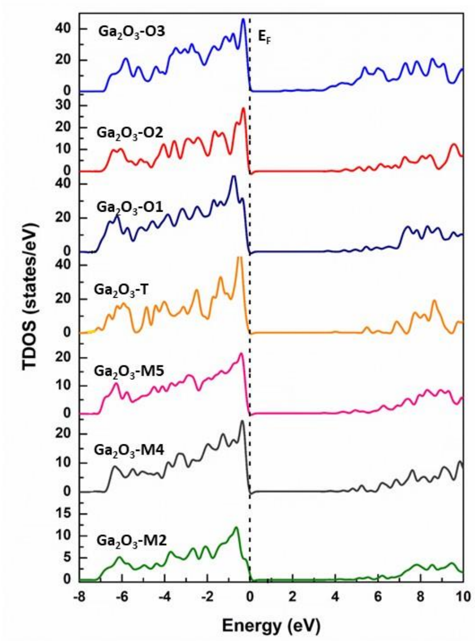

Figure S7: Total density of states of stable Ga_2O_3 polytypes
